# Supplementary material for: Influence of the vessel wall geometry on the wall-induced migration of red blood cells
Source: PLoS Comput Biol. 2023 Jul 17;19(7):e1011241. doi: 10.1371/journal.pcbi.1011241 (PMC10374106; doi:10.1371/journal.pcbi.1011241)
Supplement: S1 Text — Appendix A. Numerical methods. Appendix B. Convergence study. Appendix C. Validations of blood flow and the membrane elasticity models. Appendix D. Effect of the vessel wall with the RBC represented as a circle. Appendix E. Computational cost for the microscopic ESL and the macroscopic ESL model. Appendix F. The mobility tensor. (PDF) [file pcbi.1011241.s001.pdf]

Supporting information

Appendix A. Numerical methods.

Finite difference approximation for spatial discretization. In what follows, we employ a modified staggered-grid spatial discretization for the equations of blood developed by [13]. We assume Ω is a rectangle of size L_x by L_y discretized into an $N_x \times N_y$ rectangular grid with grid spacing $\Delta x = L_x/N_x$ and $\Delta y = L_y/N_y$. The center of the $(i,j)^{\text{th}}$ rectangular grid cell is located at $\boldsymbol{x}_{i,j} = \left(\left(i+\frac{1}{2}\right)\Delta x, \left(j+\frac{1}{2}\right)\Delta y\right)$, where $i = 0,\ldots,N_x-1$ and $j = 0,\ldots,N_y-1$. The pressure $p(\boldsymbol{x},t)$ is defined at the centers of the rectangular grid cells, and the values of p on the grid are denoted by $p_{i,j}(t) = p(\boldsymbol{x}_{i,j},t)$. We denote by $\boldsymbol{u}_{i,j}(t)$ the fluid velocities at time t and $\boldsymbol{f}_{i,j}$ the external force of the $(i,j)^{\text{th}}$ grid cell and both are located at the bottom-left corner of the $(i,j)^{\text{th}}$ grid cell (see Fig 1).

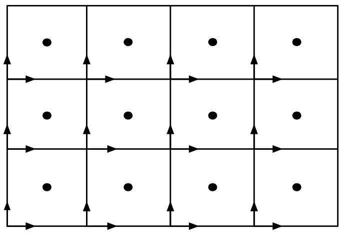

Fig 1. An illustration of the staggered-grid spatial discretization in the present work. The fluid velocities $\boldsymbol{u} = (u,v)$ are defined in terms of those vector components that are normal to the edges of the grid cells, and the pressure p is defined at the centers of the grid cells.

The spatial differential operators in Eq 1 are discretized using a second-order accurate scheme as in [11]

$$\begin{aligned} \operatorname{Re}\frac{d\boldsymbol{u}_{i,j}}{dt} + (\boldsymbol{G}p)_{i,j} &= (L\boldsymbol{u})_{i,j} + \boldsymbol{f}_{i,j} \\ (\boldsymbol{D}\cdot\boldsymbol{u})_{i,j} &= 0. \end{aligned} \tag{17}$$

The gradient of p is approximated at the cell corner by

$$(\boldsymbol{G}p)_{i,j} = \frac{1}{2}\begin{bmatrix} \frac{p_{i,j}-p_{i-1,j}+p_{i,j-1}-p_{i-1,j-1}}{\Delta x} \\ \frac{p_{i,j}-p_{i,j-1}+p_{i-1,j}-p_{i-1,j-1}}{\Delta y} \end{bmatrix}, \tag{18}$$

and the divergence of $\boldsymbol{u}$ is approximated by

$$(\boldsymbol{D}\cdot\boldsymbol{u})_{i,j} = \frac{1}{2}\left(\frac{u_{i+1,j}-u_{i,j}+u_{i+1,j+1}-u_{i,j+1}}{\Delta x} + \frac{v_{i,j+1}-v_{i,j}+v_{i+1,j+1}-v_{i+1,j}}{\Delta y}\right). \tag{19}$$

The Laplacian of $\boldsymbol{u}$ is approximated via centered difference method as

$$(L\boldsymbol{u})_{i,j} = \begin{bmatrix} \left(u_{i-1,j}-2u_{i,j}+u_{i+1,j}\right)/\Delta x^2 \\ \left(v_{i,j-1}-2v_{i,j}+v_{i,j+1}\right)/\Delta y^2 \end{bmatrix}. \tag{20}$$

The object immersed in Ω is parametrized by the Lagrangian coordinate $q \in [0, L_q]$ and is discretized using N_q points with grid spacing $\Delta q = L_q/N_q$. We denote by $\boldsymbol{X}_k(t)$ the kth Lagrangian points in Eulerian coordinates and is defined as $\boldsymbol{X}_k(t) = \boldsymbol{X}(k\Delta q, t)$, $k = 0, 1, \dots, N_q - 1$.

To obtain the velocity of each immersed boundary point using Eqs 12-13, we need the discrete delta function, $\delta_h(\boldsymbol{x})$, for interpolating and spreading. In the present work, we use the standard 4-point delta function, $\delta_h(\boldsymbol{x}) = \frac{1}{\Delta x}\phi\left(\frac{x}{\Delta x}\right)\frac{1}{\Delta y}\phi\left(\frac{y}{\Delta y}\right)$ [5], in which ϕ is given by

$$\phi(r) = \begin{cases} \frac{1}{8}(5 + 2r - \sqrt{-7 - 12r - 4r^2}), & -2 \le r < -1, \\ \frac{1}{8}(3 + 2r + \sqrt{1 - 4r - 4r^2}), & -1 \le r < 0, \\ \frac{1}{8}(3 - 2r + \sqrt{1 + 4r - 4r^2}), & 0 \le r < 1, \\ \frac{1}{8}(5 - 2r - \sqrt{-7 + 12r - 4r^2}), & 1 \le r \le 2, \\ 0, & |r| > 2, \end{cases} \tag{21}$$

and the resulting discretized immersed boundary velocity is given by

$$\boldsymbol{U}_k(t) = \sum_{i=0}^{N_x-1}\sum_{j=0}^{N_y-1} \boldsymbol{u}_{i,j}(t)\delta_h(\boldsymbol{x}_{i,j} - \boldsymbol{X}_k(t))\Delta x\Delta y, \tag{22}$$

and the external force at each Eulerian grid point is given by

$$\boldsymbol{f}_{i,j}(t) = \sum_{k=0}^{N_q-1} \boldsymbol{F}_k(t)\delta_h(\boldsymbol{x}_{i,j} - \boldsymbol{X}_k(t))\Delta q, \tag{23}$$

where the Lagrangian force at the immersed boundary points, $\boldsymbol{F}_k$, is obtained using $\boldsymbol{X}_k(t)$ through energy functions (see Eqs 10 – 12).

Implementing tether points. The implementation of tether points introduce tether forces to blood flow. The discrete external force appeared in Eq 17 is given by

$$\boldsymbol{f}_{i,j}(t) = \boldsymbol{f}_{i,j}^{\text{RBC}}(t) + \boldsymbol{f}_{i,j}^{\text{tether}}(t), \tag{24}$$

where $\boldsymbol{f}_{i,j}^{\text{IB}}(t)$ is interpolated from the Lagrangian force induced by the RBC and the ESL (Eq 8) and $\boldsymbol{f}_{i,j}^{\text{tether}}(t)$ is the interpolated tether force using the Lagrangian tether force given by Eq 10

$$\boldsymbol{f}_{i,j}^{\text{tether}}(t) = \sum_{l=0}^{N_l-1} \boldsymbol{F}_l^{\text{tether}}(t)\delta_h(\boldsymbol{x}_{i,j} - \hat{\boldsymbol{X}}_l(t))\Delta q, \tag{25}$$

Imposing physical boundary conditions. The discrete spatial differential operators (Eqs 18-20) may require modifications near physical boundaries. To simplify the presentation, we restrict our attention to the domain boundary in the vicinity of the grid cell $(0, j)$, $0 \le j \le N_y - 1$ that locates along the left side of Ω. On the staggered grid implemented in the present work, both u and v are specified on the boundaries of Ω. In this case, no boundary condition for the pressure is needed. As such no expressions are required for the ghost values $p_{0,j-1}$, $u_{0,j-1}$ or $v_{0,j-1}$.

The discrete spreading and interpolating operator $\delta_h(\boldsymbol{x})$ also have to be modified near the domain boundary, in particular, when the support of $\delta_h(\boldsymbol{x})$ overlaps with one boundary. One choice introduced in [18] is to modify $\phi(r)$ and construction a new $\delta_h(\boldsymbol{x})$ near the boundary. Here we chose another approach implemented in [19]. We use the standard 4-point delta function [5] for spreading and interpolating but extend the

domain with sufficiently many ghost cells so that the support of all delta functions is strictly within the extended domain. The ghost values of $u_{0,-1}$ and $v_{0,-1}$ are the mirror inversion of $u_{0,1}$ and $v_{0,1}$ respectively.

Discrete energy functions. The RBC immersed in Ω is discretized into N_R points with spacing $\Delta q = L_R/N_R$, and the positions of Lagrangian points in Eulerian coordinates are given by $\boldsymbol{X}_k(t) = \boldsymbol{X}(k\Delta q, t)$, $k = 0, 1, \dots, N_R - 1$. We use a bead and spring model to represent the RBC with nodes connected by Hookean springs. Discretizing the elastic energy for stretching/compressing (Eq 5) we have

$$E_{\text{spring}} = \frac{k_{\text{s}}}{2} \sum_{i=0}^{N_R-1} \left(\frac{\|\boldsymbol{X}_{i+1} - \boldsymbol{X}_i\|}{\Delta q} - 1 \right)^2 \Delta q. \tag{26}$$

We followed the approach in [4] to define the discrete bending energy as

$$E_{\text{bend}} = \frac{k_{\text{b}}}{2} \sum_{i=0}^{N_R-1} \left(\frac{\|\boldsymbol{X}_{i+1} - 2\boldsymbol{X}_i + \boldsymbol{X}_{i-1}\|}{\Delta q^2} \right)^2 \Delta q. \tag{27}$$

The discrete area-preserving penalty energy, analogous to the one used in [4], is

$$E_{\text{area}} = \frac{k_{\text{a}}}{2} \left(\sum_{i=0}^{N_R-1} \left(\frac{\boldsymbol{X}_i \times \boldsymbol{X}_{i+1}}{2} \right) - A_0 \right)^2, \tag{28}$$

where the discrete area of the cell at time t is obtained by summing up the signed area of all the triangles that are used to discretize the cell. Here the cross product corresponds to the one in 2D, given by

$$\boldsymbol{A} \times \boldsymbol{B} = A_x B_y - A_y B_x,$$

where A_x, A_y, B_x and B_y are the x and y components of vectors $\boldsymbol{A}$ and $\boldsymbol{B}$. In Eqs 26-28 indices are computed modulo N_R.

We assume that each vessel wall is discretized into N_E Lagrangian points having spacing $\Delta\hat{q}$, and the positions of Lagrangian points in Eulerian coordinates are given by $\hat{\boldsymbol{X}}_k(t) = \boldsymbol{X}(k\Delta\hat{q}, t)$, $k = 0, 1, \dots, N_E - 1$. In the microscopic ESL model, in which the ESL is described as a collection of fiber bundles, each fiber is discretized into Lagrangian points connected by elastic springs with rest length. The corresponding discrete elastic spring energy is identical to Eq 26.

Biconcave-shaped RBC. In all simulations, we assumed that the RBC is initialized with a biconcave disk shape, following the parametric description given by [14]

$$\begin{aligned} x &= a\frac{\alpha}{2}(0.207 + 2.003\sin^2\psi - 1.123\sin^4\psi)\cos\psi, \\ y &= a\alpha\sin\psi\cos\varphi, \end{aligned} \tag{29}$$

where $a = 2.8\mu$m is the equivalent cell radius, $\alpha = 1.38581894$ is the ratio between the maximum radius of the biconcave disk in the transverse plane of symmetry and a. The parameter ψ ranges from $-\pi/2$ to $\pi/2$ and the meridional angle, φ, ranges from 0 to 2π.

Appendix B. Convergence study. As a simple control for convergence, we consider the microscopic ESL model and computed the drag and lift force averaged over four initial conditions as reported previously for a fixed wall thickness, spatial variation, and permeability as the size of the mesh is successively halved. Here we fixed the permeability to be 0, the thickness of the wall to be 0.84μm, and picked an extreme case in which the density of the bundles is the highest. Using the domain $[0, 2] \times [0, 1]$, we

begin with a relative coarse 64×32 Cartesian grid, which corresponds to a mesh size of $h = 1/32$, and subsequently refined the grid obtain $h = 1/64, 1/128, 1/256$. Note that $h = 1/128$ corresponds to the 256×128 Cartesian grid used in this work.

We estimated $|\langle\text{Drag}\rangle|$ and $|\langle\text{Lift}\rangle|$ numerically by averaging over time and four initial conditions that uniformly sampled the distance between the roots of neighboring bundles. We demonstrate in Fig 2 as the mesh width h is successively halved, both $|\langle\text{Drag}\rangle|$ and $|\langle\text{Lift}\rangle|$ converge. In both cases, the convergence starts around $h = 1/128$.

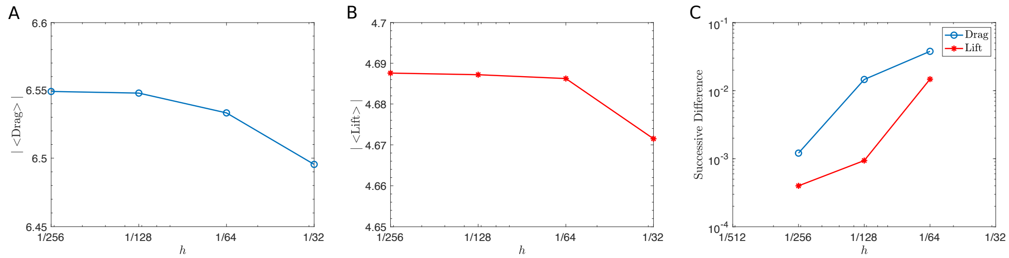

Fig 2. Convergence study using the microscopic ESL model with a sequence of refined grids. (A and B) Time and initial condition averaged drag and lift force as the mesh size is successively halved. (C) The difference between successive points on the $|\langle\text{Drag}\rangle|$ vs h and $|\langle\text{Lift}\rangle|$ vs h curves from (A) and (B). The smaller of the two h values is used for labeling. For all simulations $T = 50$s.

Appendix C. Validations of blood flow and the membrane elasticity models. As a benchmark, we first applied the macroscopic ESL model (described in Results) with dimensional parameters to the problem setup described in [3] focusing on the effects of porous wall layer on the motion of a single deformable membrane near microvessel walls. We examine two scenarios corresponding to impermeable ESL layers and permeable layers with a hydraulic resistivity of $\kappa = 10^{11}\text{N}\cdot\text{s/m}^4$ [3]. For impermeable layers, the computational domain is a rectangular channel of 16μm in length and 8μm in width equipped with a periodic boundary condition in the horizontal direction and a no-slip boundary condition in the vertical direction. In this case, ESLs are modeled explicitly as solid walls and tether points are not used. For permeable layers, the computational domain is a rectangle of length 20μm and width 10μm with the same boundary conditions as in the impermeable case. An ESL of width 1 μm is assumed and is modeled as a straight line as in [2,3]. In both cases the initial configuration of the membrane is a circle of radius 2.66 μm and is placed with its center 0.9 μm from the center-line of the channel. A body force of the form described above in Eq 4 is applied and the magnitude is chosen so that the maximum flow velocity matches the one in [3] (approximately 0.8mm/s) in the absence of the membrane with impermeable ESLs.

One fundamental difference between our approach and the one used in [3] is the model of permeability of the layer. In [3] the porous ESL is modeled using Brinkman's approximation and the variation in the layer's permeability is characterized by the hydraulic resistivity (κ). To determine the corresponding porosity constant, k_p, in our model, we estimated k_p via $\kappa = \mu/k_\text{p}$ [12,16]. Here μ is the fluid viscosity and is set to 10^{-3}Pa$\cdot$s as in [3]. We summarized in Table 1 the parameters used in our simulations.

We compute the motion and deformation and the distance of the center of mass of the membrane from the center-line of the channel and compare them to those reported in Fig 2 and Fig 3 of [3]. We show in Figs 3 and 4 that the results of our macroscopic ESL model are initially in good agreement with [3], as the membrane migrates away from the top layer and moves towards the center-line of the channel. After 200ms however, the membrane continuously moves towards the center-line and reaches a steady

Table 1. Parameters for Figs 3 and 4.

Parameter	Description	Value
Re	Reynolds number	0.01 [1, 11]
$k_{\rm s}^{\rm RBC}$	Elastic spring constant	3 μN/m [14]
$k_{\rm b}$	Bending constant	2×10^{-19} N·m [4, 17]
$k_{\rm a}$	Area preserving constant	185 N/μm^2
$k_{\rm tether}$	Tether force constant	3200 N/μm
$k_{\rm p}$	Porosity constant	0, 0.01μm^2 [3]
Δx, Δy	Domain mesh spacing	0.0078μm
Δq	Lagrangian mesh spacing	0.0039μm
Δt	Time-step size	0.0001s

state in our model whereas in [3] the center of mass of the membrane appears to oscillate slightly (see Fig 4). In comparing the deformation of the membrane between the impermeable and permeable case, we find that, similar to [3], the shape of the membrane remains largely unchanged using our macroscopic ESL model (see Fig 3). We note that the deformation of the membrane using our two-dimensional model differs somewhat from that reported in [3], in that the parachute-like concavity on the trailing end of the membrane is less prominent in our simulations than in [3]. However, note that the resulting shapes are not expected to closely resemble those of a flowing RBC since the membrane is initialized as a circle in this simulation, whereas the equilibrium configuration of an RBC is known to be a biconcave disk, which has a significantly smaller reduced volume. While the circular reference configuration is satisfactory for benchmarking our simulation setup in the context of a deformable membrane, when simulating RBC's in the following sections we will use a biconcave disk initial configuration.

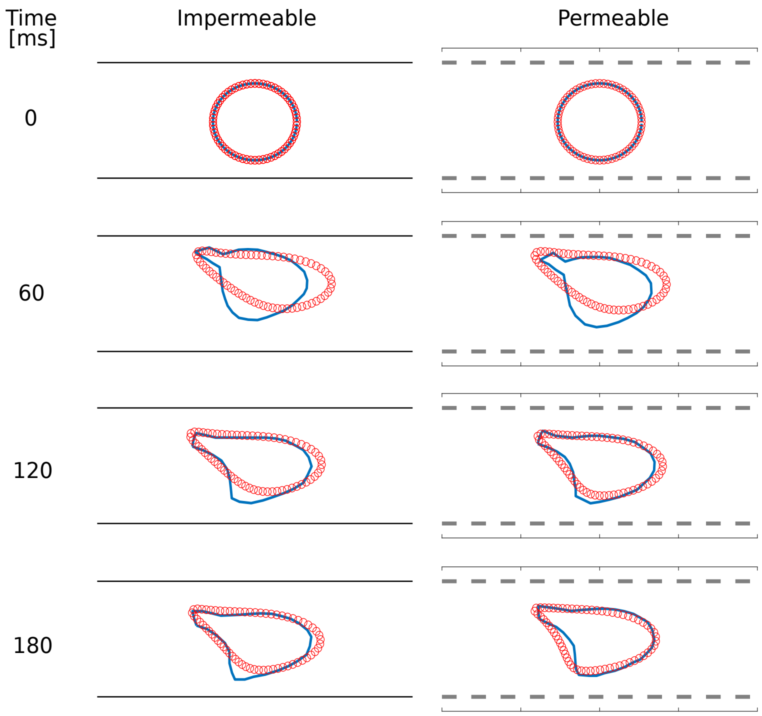

Fig 3. Motion and deformation of the RBC. The RBC is initially placed at 0.9 μm from the center-line with snapshots taken at 60-ms intervals for the two cases as indicated. Results extracted from [3] using [15] are plotted in solid blue lines. Results of using our macroscopic ESL model are plotted in red circles. The dashed gray lines indicate the location of permeable ESLs. Parameters are summarized in Table 1.

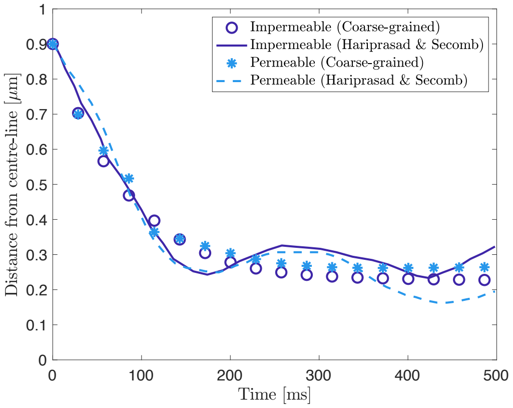

Fig 4. Distance of center of mass of the RBC from the center-line of the channel. Results extracted from [3] using [15] are plotted in solid (impermeable) and dashed (permeable, $\kappa = 10^{11}$N·s/m^4) lines. Results of using our macroscopic ESL model are plotted in circles (impermeable) and stars (permeable, $k_\mathrm{p} = 10^{-2}\mu$m^2). Parameters are summarized in Table 1.

Appendix D. Effect of the vessel wall with the RBC represented as a circle. When modeling the RBC in 2D, the shape of the RBC is frequently represented as a circle [2, 3]. To demonstrate that our choice of the 30 degree initial orientation of the biconcave-shaped RBC does not alter the simulation result, we replaced the RBC by a circle of the same area as the biconcave-shaped RBC used in the study and repeated the simulations for a selected sets of parameter values using both the microscopic ESL and the macroscopic ESL model.

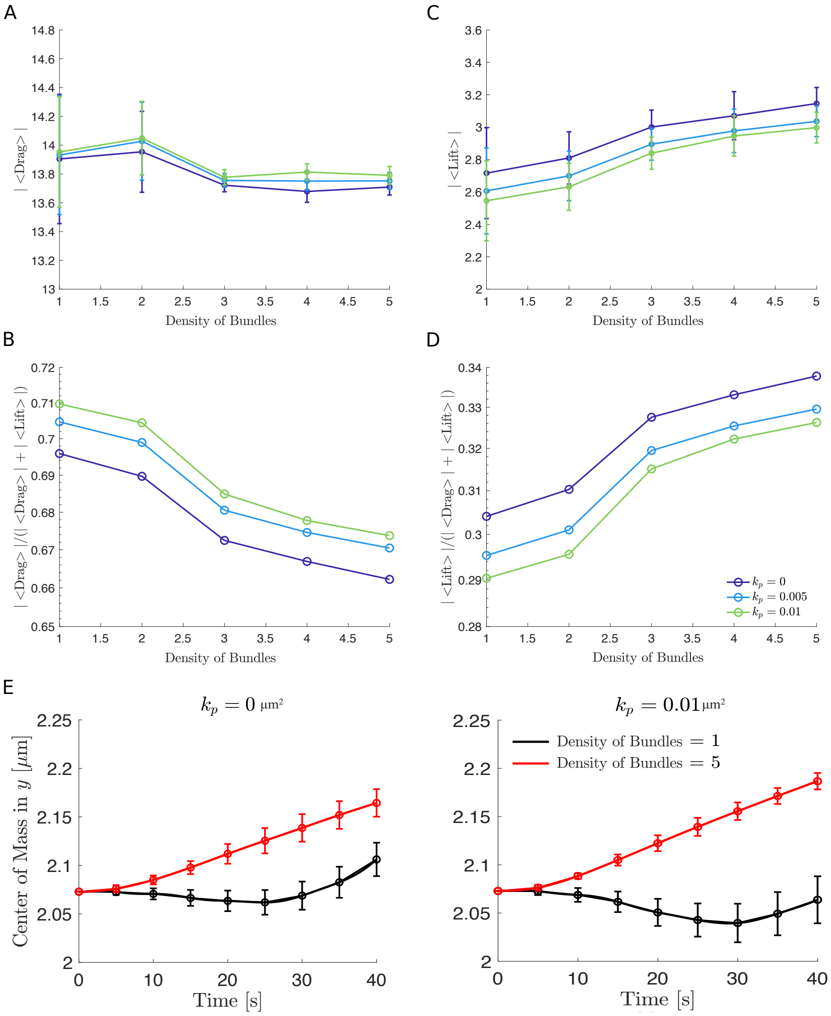

Fig 5. Effect of spatial variation using a circle in the microscopic ESL model. (A and C) Time and initial condition averaged drag and lift force versus layer spatial density. (B and D) Time and initial condition averaged fraction of drag ($|\langle\text{Drag}\rangle| / (|\langle\text{Drag}\rangle| + |\langle\text{Lift}\rangle|)$) and lift force ($|\langle\text{Lift}\rangle| / (|\langle\text{Drag}\rangle| + |\langle\text{Lift}\rangle|)$) versus spatial density over three permeability values as indicated. (E) The RBC's center of mass in the y direction versus time for an impermeable wall and a highly permeable wall. In all simulations, the thickness is fixed to be $h = 1.4839\mu$m. In panel A, C, and E 95% confidence intervals are plotted at each data point. Parameters are summarized in Table 2 in Results.

We analyzed the motion of the circular RBC by estimating $|\langle\text{Drag}\rangle|$ and $|\langle\text{Lift}\rangle|$ numerically by averaging over time and four initial conditions. In comparing Figs 5 and 6 to Figs 4 and 9 in Results, we see that the effect of spatial variation using the circle

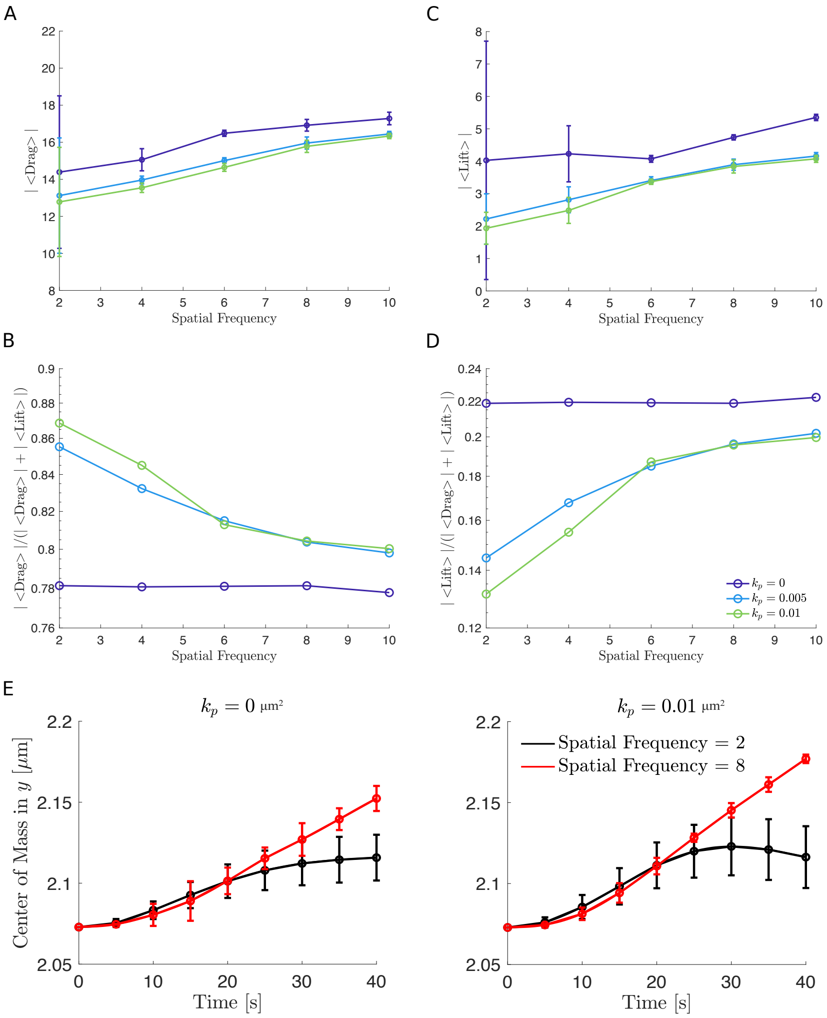

Fig 6. Effect of spatial variation using a circle in the macroscopic ESL model. (A and C) Time and initial condition averaged drag and lift force versus layer spatial frequency. (B and D) Time and initial condition averaged fraction of drag $(|\langle \text{Drag}\rangle| / (|\langle \text{Drag}\rangle| + |\langle \text{Lift}\rangle|))$ and lift force $(|\langle \text{Lift}\rangle| / (|\langle \text{Drag}\rangle| + |\langle \text{Lift}\rangle|))$ versus spatial frequency over three permeability values as indicated. (E) The RBC's center of mass in the y direction versus time for an impermeable wall and a highly permeable wall. In all simulations, the thickness is fixed to be $(A+h) = 1.4839\mu$m. In panel A, C and E 95% confidence intervals are plotted at each data point. Parameters are summarized in Table 1 in Results.

agrees with the ones using a biconcave disk with a 30 degree initial orientation qualitatively in both the microscopic ESL and the macroscopic ESL model. That is, the motion of the RBC is affected more by the changes in the spatial variation when the ESL is highly permeable. Comparing Figs 7 and 8 to Figs 7 and 11 in Results, we see that the effect of varying wall thickness again agrees with the ones using a biconcave disk qualitative in both models. The thicker the wall, the harder the RBC could move away from the wall.

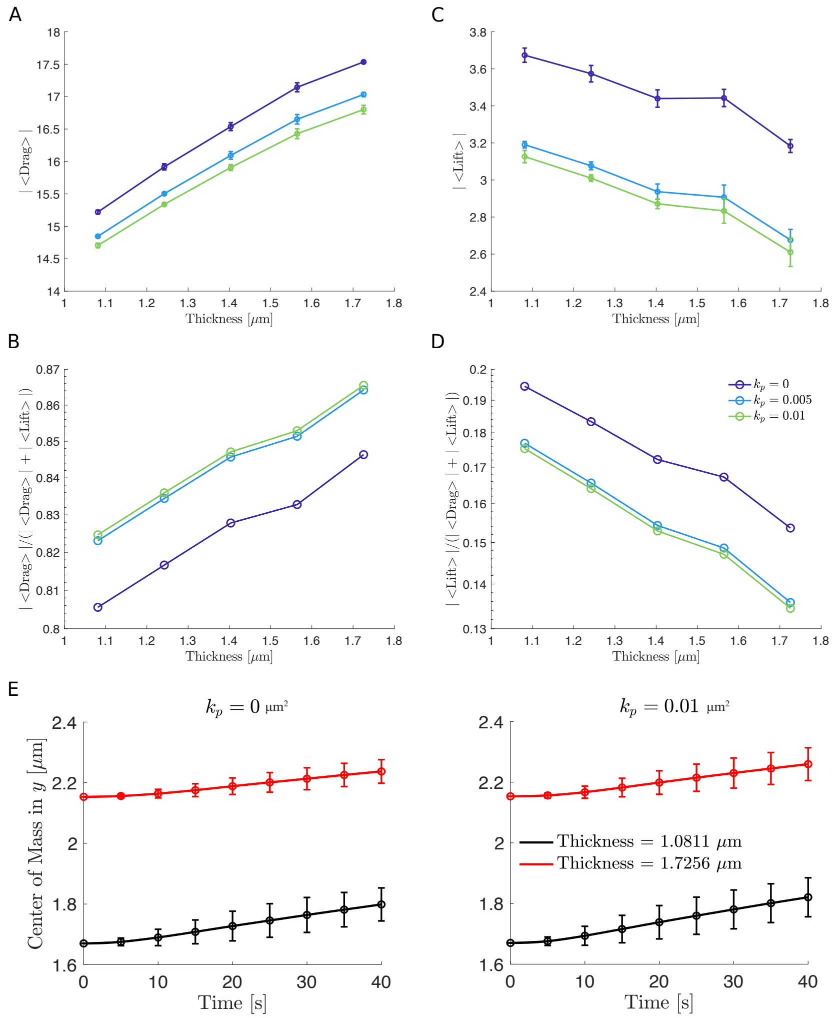

Fig 7. Effect of thickness using a circle in the microscopic ESL model. (A and C) Time and initial condition averaged drag and lift force versus layer thickness. (B and D) Time and initial condition averaged fraction of drag ($|\langle\text{Drag}\rangle| / (|\langle\text{Drag}\rangle| + |\langle\text{Lift}\rangle|)$) and lift force ($|\langle\text{Lift}\rangle| / (|\langle\text{Drag}\rangle| + |\langle\text{Lift}\rangle|)$) versus thickness over three permeability values as indicated. (E) The RBC's center of mass in the y direction versus time for an impermeable wall and a highly permeable wall. we assume the ESL is in a healthy condition corresponding to a spatial frequency of 1. In panel A, C and E 95% confidence intervals are plotted at each data point. Parameters are summarized in Table 1 in Results.

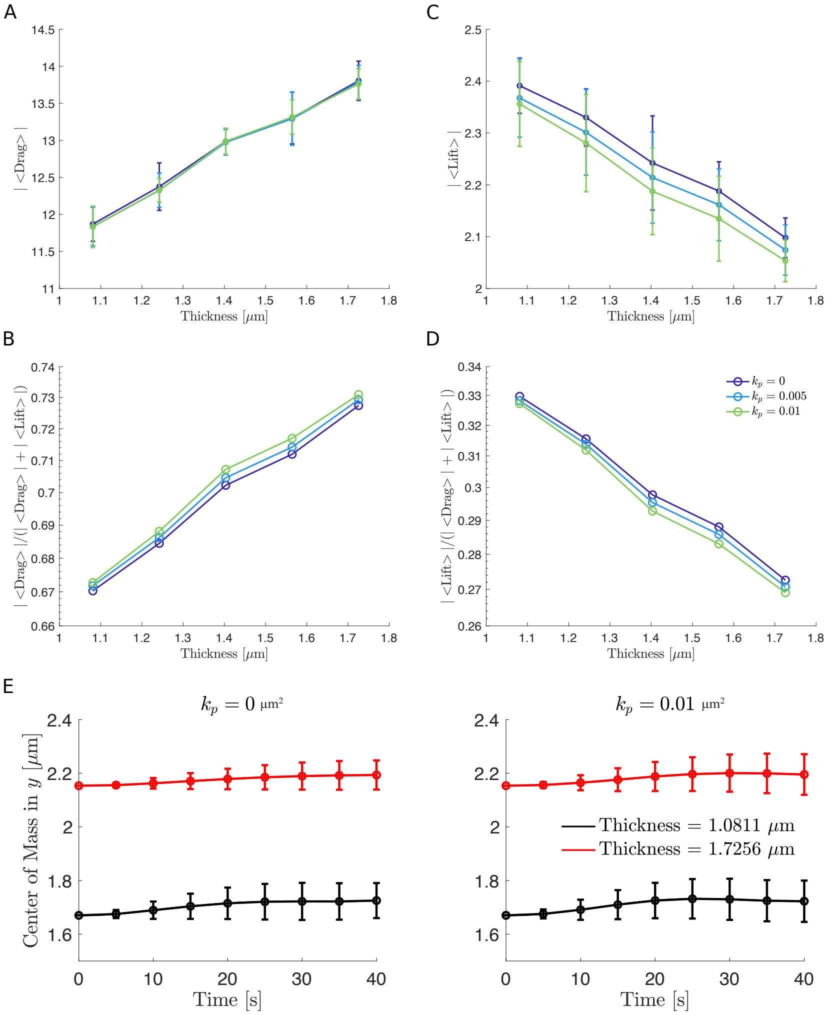

Fig 8. Effect of thickness using a circle in the macroscopic ESL model. (A and C) Time and initial condition averaged drag and lift force versus layer thickness. (B and D) Time and initial condition averaged fraction of drag ($|\langle \text{Drag} \rangle| / (|\langle \text{Drag} \rangle| + |\langle \text{Lift} \rangle|)$) and lift force ($|\langle \text{Lift} \rangle| / (|\langle \text{Drag} \rangle| + |\langle \text{Lift} \rangle|)$) versus thickness over three permeability values as indicated. (E) The RBC's center of mass in the y direction versus time for an impermeable wall and a highly permeable wall. we assume the ESL is in a healthy condition corresponding to a spatial frequency of 1. In panel A, C and E 95% confidence intervals are plotted at each data point. Parameters are summarized in Table 2 in Results.

Appendix E. Computational cost for the microscopic ESL and the macroscopic ESL model. As a simple benchmark and comparison for the computational cost of the microscopic ESL and the macroscopic ESL model, we fixed the thickness of the ESL to be 0.84μm and picked a relative extreme case in which both the density of the bundles (microscopic ESL) and the spatial frequency (macroscopic ESL) are high. We examined three different values of $k_{\mathrm{p}} = 0, 0.005$, and $0.01\mu\mathrm{m}^2$ with each case simulated till $T = 50$s.

To record the computational time for each case, we made use of MATLAB's built-in timer `tic` and `toc`. For all three values of k_{p}, the macroscopic ESL model is approximately 2 times faster than the microscopic ESL model and the macroscopic ESL model becomes more computationally efficient as k_{p} is increased (see Table 2).

Table 2. Comparison of the computational cost of the detailed and coarse-grained model

	Microscopic ESL	Macroscopic ESL
$k_{\mathrm{p}} = 0\mu\mathrm{m}^2$	5151s	2842s
$k_{\mathrm{p}} = 0.005\mu\mathrm{m}^2$	5357s	2844s
$k_{\mathrm{p}} = 0.01\mu\mathrm{m}^2$	5360s	2849s

Appendix F. The mobility tensor. For a spherical particle immersed in low-Reynolds number flow contained by rigid boundaries, its velocity, $\boldsymbol{U}$ and the force acting on the particle, $\boldsymbol{F}$, can be related through

$$\boldsymbol{U} = \mathcal{M} \cdot \boldsymbol{F},$$

where $\mathcal{M}$ is the mobility tensor that is symmetric, positive-definite, and for a given force yields the particle motion parallel and perpendicular to the wall. In three dimensions, components of the mobility tensor have been studied using series approximation [6–9]. In two dimensions, one can think of the problem as an infinite cylinder in three-dimensional low-Reynolds number flow [10]. The component of $\mathcal{M}$ that corresponds to the parallel direction to the wall is given by

$$\mathcal{M}_D = -\frac{\log\left(\frac{h+\sqrt{h^2+r^2}}{r}\right)}{4\pi\mu},$$

whereas the component of $\mathcal{M}$ that corresponds to the direction normal to the wall is given by

$$\mathcal{M}_L = -\frac{\log\left(\frac{h+\sqrt{h^2+r^2}}{r} - \frac{\sqrt{h^2-r^2}}{h}\right)}{4\pi\mu}.$$

Here h denotes the distance of the cylinder axis to the wall, r denotes the radius of the cylinder, and μ is the viscosity of the flow.

References

1. Cantat I, Misbah C. Lift force and dynamical unbinding of adhering vesicles under shear flow. Physical Review Letters. 1999; 83(4):880.

2. Secomb TW, Hsu R, Pries AR. A model for red blood cell motion in glycocalyx-lined capillaries. American Journal of Physiology – Heart and Circulatory Physiology. 1998; 274(3):H1016-H1022.

3. Hariprasad DS, Secomb TW. Motion of red blood cells near microvessel walls: effects of a porous wall layer. J. Fluid. Mech. 2012; 705:195–212.

4. Shi L, Pan T-W, Glowinski R. Numerical simulation of lateral migration of red blood cells in Poiseuille flows. Int. J. Numer. Meth. Fluids. 2012; 68:1393–1408.

5. Peskin CS. The immersed boundary method. Acta Numerica. 2002; 11:479–517.

6. Swan JW, Brady JF. Simulation of hydrodynamically interacting particles near a no-slip boundary. Physics of Fluids. 2007; 19:113306.

7. Swan JW, Brady JF. Particle motion between parallel walls: Hydrodynamics and simulation. Physics of Fluids. 2010; 22:103301.

8. Usabiaga FB, Xie X, Delgado-Buscalioni R, Donev A. The Stokes-Einstein relation at moderate Schmidt number. J. Chem. Phys. 2013; 139:214113.

9. Huang P, Guasto JS, Breuer KS. The effects of hindered mobility and depletion of particles in near-wall shear flows and the implications for nanovelocimetry. J. Fluid Mech. 2009; 637:241.

10. Jeffrey DJ, Onishi Y. The slow motion of a cylinder next to a plane wall. The Quarterly Journal of Mechanics and Applied Mathematics. 1981; 34(2):129.

11. Fai TG, Rycroft CH. Lubricated immersed boundary method in two dimensions. J. Comput. Phys. 2018; 356(1):319–339.

12. Stockie JM. Modelling and simulation of porous immersed boundaries. Computers and Structures. 2008; 87:701–709.

13. Almgren AS, Bell JB, Szymczak WG. A numerical method for the incompressible Navier-Stokes equations based on an approximate projection. SIAM J. Sci. Comput. 1996; 17(2):358–396.

14. Pozrikidis C. Numerical simulation of the flow-induced deformation of red blood cells. Annals of Biomedical Engineering. 2003; 33:1194–1205.

15. 2D Reader, graphreader. `http://www.graphreader.com`.

16. Leiderman KM, Miller LA, Fogelson AL. The effects of spatial inhomogeneities on flow through the endothelial surface layer. Journal of Theoretical Biology. 2008; 252:313–325.

17. Pozrikidis C. Modeling and Simulation of Capsules and Biological Cells. Boca Raton: Chapman & Hall/CRC; 2003.

18. Griffith BE. An accurate and efficient method for the incompressible Navier-Stokes equations using the projection method as a preconditioner. J. Comput. Phys. 2018; 228(20):7565–7595.

19. Kallemov B, Bhalla APS, Griffith BE, Donev A. An immersed boundary method for rigid bodies. Comm. App. Math. and Comp. Sci. 2016; 11(1):79–141.
