# Supplementary material for: Influence of the vessel wall geometry on the wall-induced migration of red blood cells
Source: PLoS Comput Biol. 2023 Jul 17;19(7):e1011241. doi: 10.1371/journal.pcbi.1011241 (PMC10374106; doi:10.1371/journal.pcbi.1011241)
Supplement: Attachment — Submitted filename: Resp_to_rev_PLOS.pdf [file pcbi.1011241.s008.pdf]

Dear PLOS Comp. Biol. Editor,

Thank you very much for your consideration of our manuscript. We thank the reviewers for their helpful comments and suggestions. Major changes to address the reviewers comments include:

1. Expanding the Introduction section with the biology background, motivation, and explanation of what has been done using mathematical modeling approaches and contribution of this work.
2. Reorganizing the Results section by moving the first subsection to appendix so that the Results section begins with the microscopic ESL model (previously called the detailed model) followed by the macroscopic ESL model (previously called the coarse-grained model) with applications of the macroscopic ESL model.
3. Adding new figures showing RBC positions for a selected set of parameter values.
4. Adding new simulation results using a circle to represent the RBC, serving as a validation/comparison of our choice of the 30 degree initial orientation for the biconcave-shaped RBC.
5. Expanding the Discussion section to discuss the choice of 2D models, their limitations, and future directions.

Please find the attached updated manuscript with changes in blue. We have provided detailed responses to the reviewers' comments below and in the updated text.

Sincerely,

Ying Zhang and Thomas Fai

Reviewer #1:

To first discuss positives, the authors have made their code available via github. The topic (the effects of ESL properties on the width of the cell free layer, which affects flow resistance in vessels, which affects blood flow and transport in general) does not yet seem to be the subject of others. It is also interesting to note that even though the red blood cells do not directly interact with the structural components of the ESL, the properties of those structural components (particularly thickness) can affect the cell free layer. The attempt to use experimental data was also refreshing to see though the implementation was not completely clear to the reviewer.

Response:
We appreciate the reviewers' positive feedback, including our attempt to interpret available experimental data in light of our model. We clarify the implementation in the comments below. The purpose of using the experimental data is to:
1. Demonstrate the ability of our model for handling the ESL geometries that appear in a more realistic setting, which does not appear in previous studies.
2. Provide some insights about the influence of the ESL on the microcirculation (here we focused on analyzing the width of the cell-free layer) using actual images taken from experiments. In particular, we would like to understand if the estimated width of the cell-free layer obtained using the experimental data would differ significantly from those reported in the literature, estimated from more idealized settings, such as glass tubes (see Fig 15 in the manuscript).
We further clarify the reason for using experimental data in the introduction (see lines 64-67).

While the reviewer appreciates the amount of work put into the paper, there are several concerns that the reviewer was hoping could be addressed. Some of the more major concerns include: A more thorough introduction including motivation and what others have done; A better explanation of the methods used in the study; Using cell position rather than drag and lift as the primary assessment tools during the study; more clearly taking into account multiple potentially confounding factors; and more clearly explaining counterintuitive outcomes. The use of the drag and lift to support results regarding the cell free layer (aka average distance of red blood cells from the wall) rather than more direct red blood cell position estimates make some of these results difficult to believe. For example, the authors state increased drag "hinders the migration of the RBC" towards the centerline even though drag does not push the cell towards or away from the centerline. Drag only acts along the centerline, not towards or away from it.

For the introduction, the authors mention the cell-free layer (CFL) and imply that it is important but never really say why. Related, how might the ESL affect that CFL and related downstream important events? The introduction could also use a better discussion of what's been done before and how the author's are presenting a unique contribution. For instance, there is a very similar study by Hariprasad and Secomb that the authors use the data from but they don't discuss the results, importance, and differences (vs. their study) of that study in the introduction. Another example is the use of 0.5 microns for the outer layer of the endothelial glycocalyx. The reference is from a 2007 Weinbaum paper while other estimates have been made since then

(see e.g. works by Fitzroy Curry). In general, it feels like the literature review could and should be a little more extensive.

Response:
We agree with the reviewer that the manuscript will be further improved by a more thorough literature review. We significantly expanded the introduction to
1. Clarify the importance of understanding CFL formation
   (a) The CFL contributes to the rheological properties of blood in arterioles and venules [12].
   (b) The CFL is shown to modulate the nitric oxide scavenging effects by RBCs [13].
   (c) The CFL enhances plasma skimming, which potentially contributes to a heterogeneous distribution of RBCs in capillaries [14-16].
2. Discuss that the presence of the ESL in microvessels is believed to be a contributing factor to the substantial increase in flow resistance and thus potentially affect the CFL formation [32].
3. Review previous studies for investigating the effects of the ESL on blood flow and the mechanics of RBC motion. Methods include lubrication theory [17,18], Brinkman approximation [48], modeling RBCs as rigid spheres [17,18], viscoelastic membrane [19,32] and a combination of viscoelastic and viscous elements [48].
4. Discuss the main differences between our computational model and existing models. In our model, the interaction between blood flow and RBC and ESL is coupled using an immersed boundary method and the permeability of the ESL is directly imposed through Darcy's law. The model offers a better flexibility of handling a more complex ESL geometry, which could be beneficial to investigate the influence of the ESL geometry using experimental data.

The reviewer had difficulty understanding how the components of the model fit together and how the study was ultimately organized. It seems like there are structural components modeled either by multiple line segments ("detailed model") or by a continuous sinusoidal curve ("coarse-grained model"). On top of that is an additional component of the model that turns the fluid into a porous media. I believe this "fluid model" is 1 micron thick thought this seems to be mentioned in just one of the 3 major sections suggesting maybe it changed in the other two studies? Additional comments on the model, particularly inaccuracies and aspects that didn't seem straightforward, are discussed later.

Response:
The reviewer is correct that we used two different representations to model the structure of the ESL from a microscopic perspective (termed the microscopic ESL model in the revision, previously the detailed model) and a macroscopic perspective (termed the macroscopic ESL model in the revision, previously the coarse-grained model). In both cases, the blood flow is modeled by the unsteady Stokes equations on a semi-periodic rectangular domain.

To make the manuscript structure clearer throughout, we renamed the "detailed model" as "microscopic ESL model" and the "coarse-grained model" as "macroscopic ESL model". We also reorganized the Results section by moving the first subsection "Validations of blood flow and the membrane elasticity model" to the appendix (see S3 Appendix) and directly started with using

the microscopic ESL model to investigate the effect of spatial variation, thickness, and permeability of the ESL using a single RBC. Then we repeated the same analysis using the more computationally efficient macroscopic ESL model to show that the results agree qualitatively with those from using the microscopic ESL model. Finally, we made use of the macroscopic ESL model with the extracted vessel wall geometry to investigate the formation of CFL under healthy and septic scenarios.

Most of the results consist of the calculation of drag and lift for these cells. While I think I recall a select few papers considering lift and drag for vesicles (I think) near walls, if the authors are really interested in the cell free layer, why not just plot/analyze the distance of the red blood cell to the wall (e.g. Fig. 3)? Such plots more directly indicate what we can expect in terms of the cell free layer. The drag points directly upstream and, unless the reviewer is missing something, tells us only about what we can expect regarding red blood cell motion upstream and downstream, not towards or away from the centerline of the vessel. I believe integrating the unsteady Stokes equation reveals that the lift is (approximately?) proportional to the average acceleration of the cell (towards the centerline) but it still seems that it is significantly easier to just to plot positions and/or velocities directly. I believe that basing assessments on the more straightforward assessor would be better for readers. I should also note that while lift can be related to the vertical acceleration of the cell, migration of cells (and vesicles) still happens in steady stokes flow where lift (and drag) is zero. Contributing migration solely to lift (e.g. line 13) is probably not a good idea (clarify if the reviewer is misunderstanding).

Response:
We appreciate the reviewer's suggestion that looking at positions of the RBC at various time steps would be helpful. To further illustrate and quantify the wall-induced migration,we have included 4 figures showing the positions of the RBC for a subset of cases examined (see Fig 2 B and C, Fig 4, Fig 7 B and C, and Fig 9, also attached below), with 95% confidence intervals obtained over four initial positions of the RBC. Note that the drag and lift force do indeed refer to motion toward or away from the centerline. That is, because the domain includes no-slip boundaries, the mobility is a tensor consisting of two elements that are parallel and perpendicular to the wall. Moreover, the corresponding components of drag depend on the distance between the cell and the wall [20-22]. In this work, we are analyzing the perpendicular force, which we termed the lift, as well as the component of force in the negative x-direction, termed drag. In the main text, we have included a discussion about the mobility tensor to clarify the definitions of the drag and lift studied in this work (see lines 88-92).

Fig 2 B and C (The distance of the RCB's center of mass to the wall for different spatial bundle density):

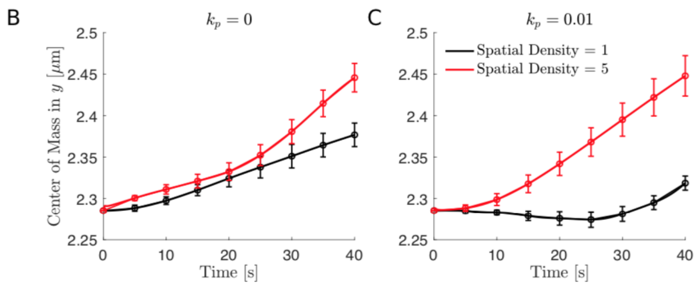

Fig 4 (The distance of the RCB’s center of mass to the wall for different thickness using the microscopic ESL model):

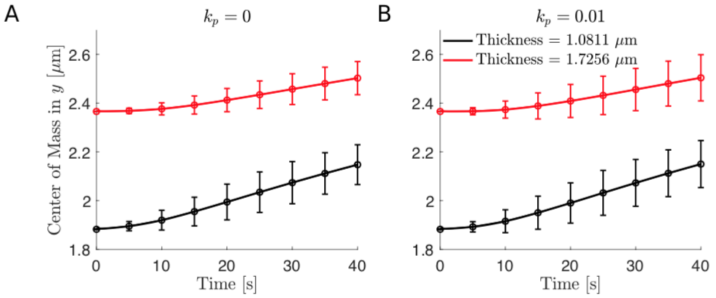

Fig 7 B and C (The distance of the RCB’s center of mass to the wall for different spatial frequency using the macroscopic ESL model):

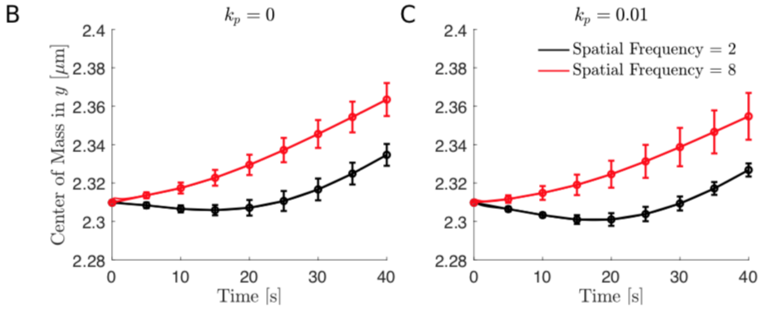

Fig 9 (The distance of the RCB's center of mass to the wall for different thickness using the macroscopic ESL model):

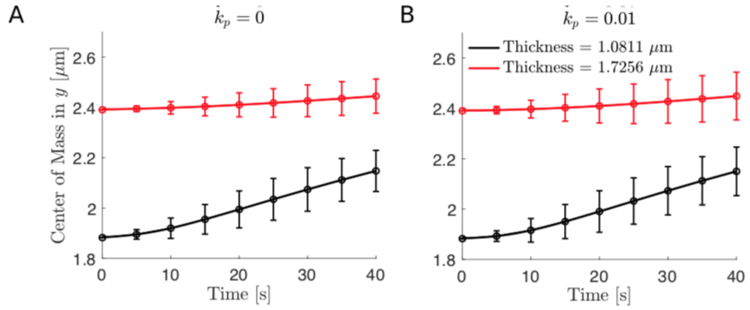

For confounding factors, it is very difficult to know what factors might be playing an important role in the results presented. It seems the authors always orient the red blood cell 30 degrees.

Results could depend on this choice and results are best averaged over all orientations. Alternatively, one could start with a circle to obtain less biased results. I feel I should note that the authors incorrectly state/imply that the proper shape for a 2D red blood cell is a biconcave disk. Biconcave disks only exist in 3D and given past 2D model validations, starting with a circle is arguably just as good and perhaps even better than the biased “biconcave” shape frequently used by 2D modelers. The experimental study seems more like a study regarding a larger vessel vs. a smaller vessel where the smaller vessel has been made smaller not necessarily due to ESL changes but due to inflammatory changes like leukocytes taking up residence at the vessel walls. I imagine that the ESL has been damaged (see, e.g., one of Curry’s responses to a recent paper on a damaged ESL) but the effects of such changes are dwarfed by the changed effective diameter. This latter study doesn’t really seem to be looking at the effects of the ESL, it seems to be looking at the effects of vessel diameter, which has been studied relatively well (as the authors note). In fact, with a bit more simulation I imagine the authors could show that the altered flow doesn’t really depend on the ESL, it depends on the diameter changes induced by inflammatory cells, which may be an interesting result.

Response:

We agree with the reviewer that ideally one would analyze the results by averaging over all possible orientations of the RBC. To study the effect of different orientations in a computationally efficient manner, we have repeated the simulations using both the microscopic ESL and the macroscopic ESL model for a selected parameter set using a circle and compared our previous results to the new simulations. We see that the results between using a circle (see S6 Fig - S9 Fig, also attached below on the left column) and the 30 initial orientation of a biconcave-shaped RBC (also attached below on the right column) agree qualitatively.

S6 Fig (Effect of spatial variation using a circle in the microscopic ESL model, on the left)

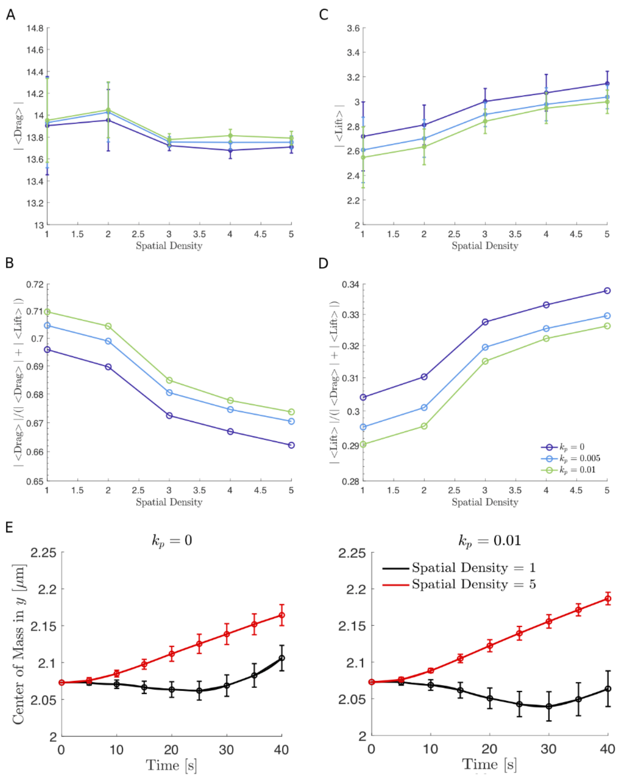
A
| <Drag> |
Spatial Density
C
| <Lift> |
Spatial Density
B
| <Drag> |/(| <Drag> | + | <Lift> |)
Spatial Density
D
| <Lift> |/(| <Drag> | + | <Lift> |)
Spatial Density
$k_p = 0$
$k_p = 0.005$
$k_p = 0.01$
E
$k_p = 0$
$k_p = 0.01$
Center of Mass in y [μm]
Time [s]
Spatial Density = 1
Spatial Density = 5

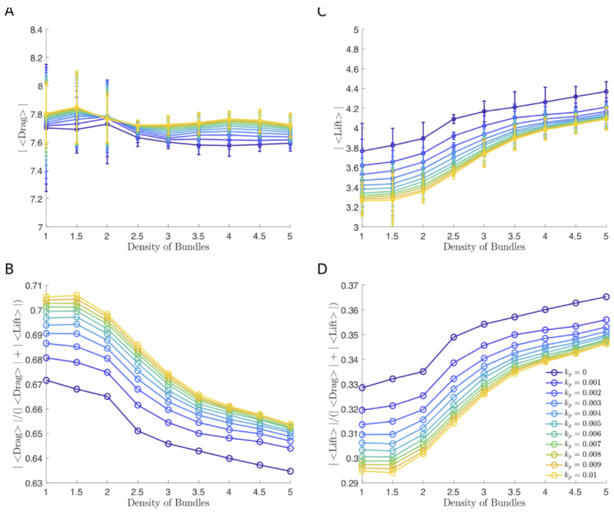
A
| <Drag> |
Density of Bundles
C
| <Lift> |
Density of Bundles
B
| <Drag> |/(| <Drag> | + | <Lift> |)
Density of Bundles
D
| <Lift> |/(| <Drag> | + | <Lift> |)
Density of Bundles
$k_p = 0$
$k_p = 0.001$
$k_p = 0.002$
$k_p = 0.003$
$k_p = 0.004$
$k_p = 0.005$
$k_p = 0.006$
$k_p = 0.007$
$k_p = 0.008$
$k_p = 0.009$
$k_p = 0.01$

S7 Fig (Effect of thickness using a circle in the microscopic ESL model):

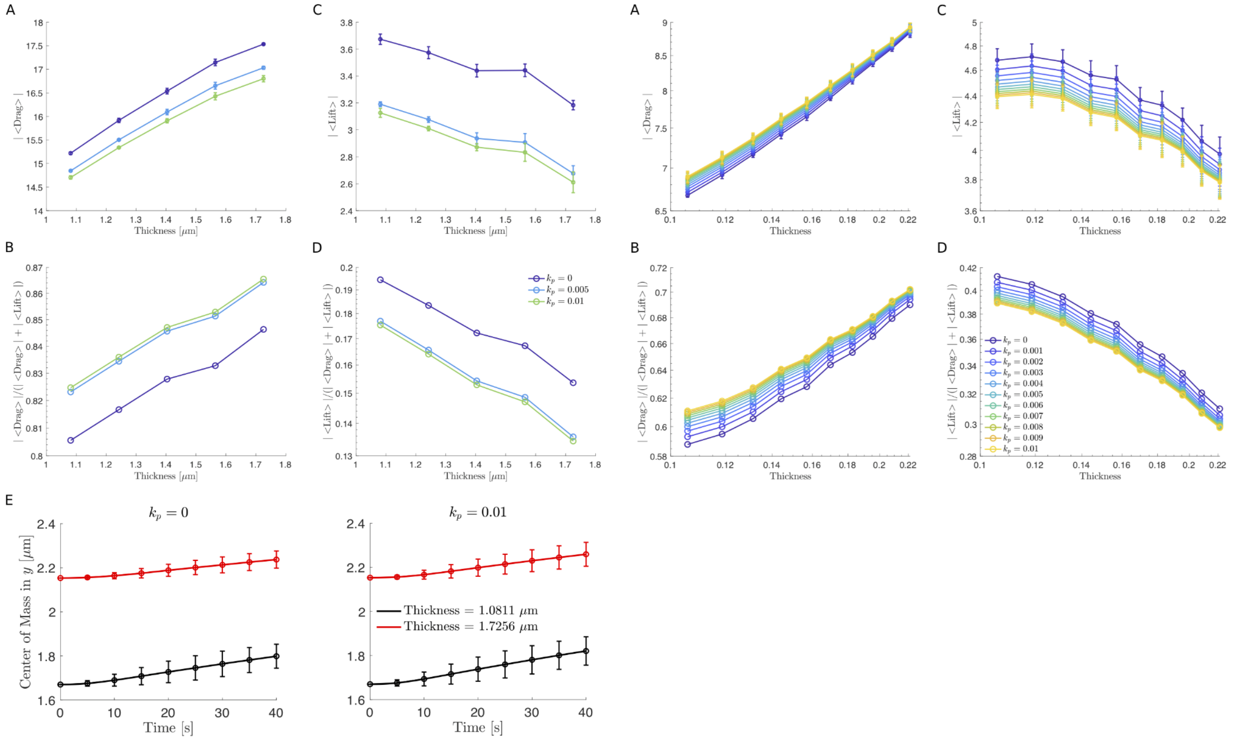

S8 Fig (Effect of spatial variation using a circle in the macroscopic ESL model

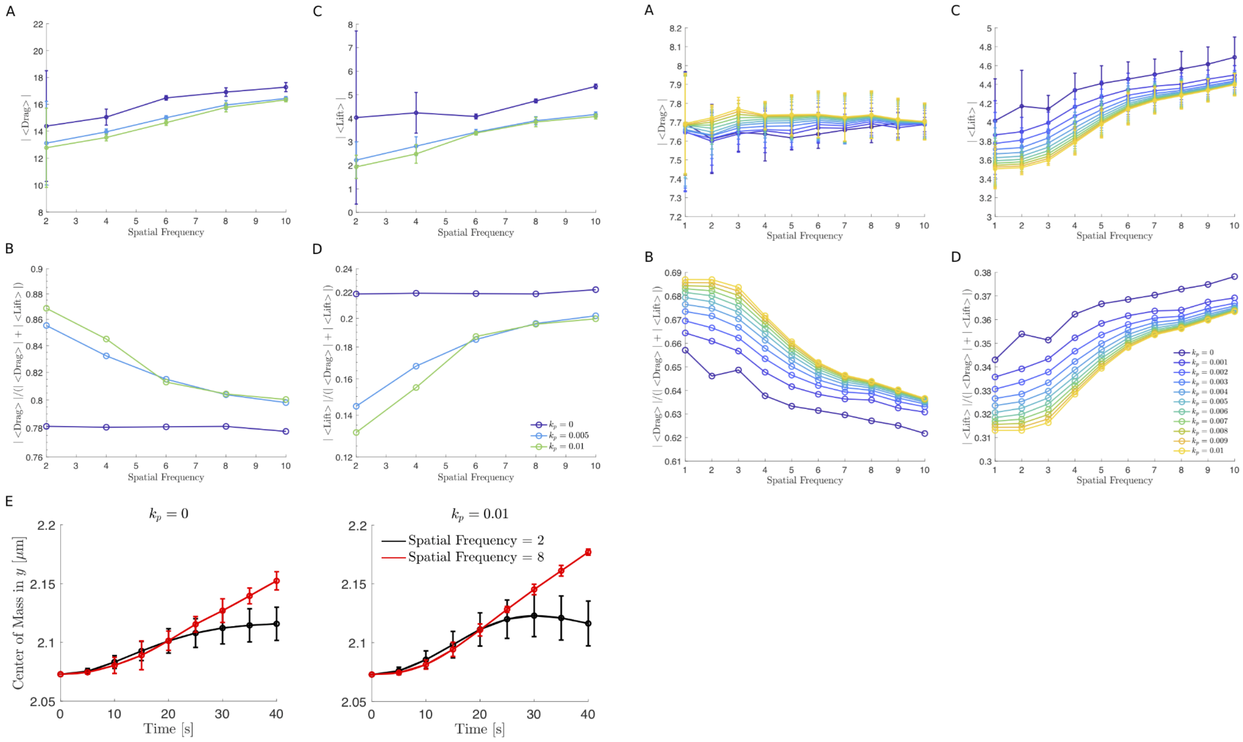

S9 Fig (Effect of thickness using a circle in the macroscopic ESL model):

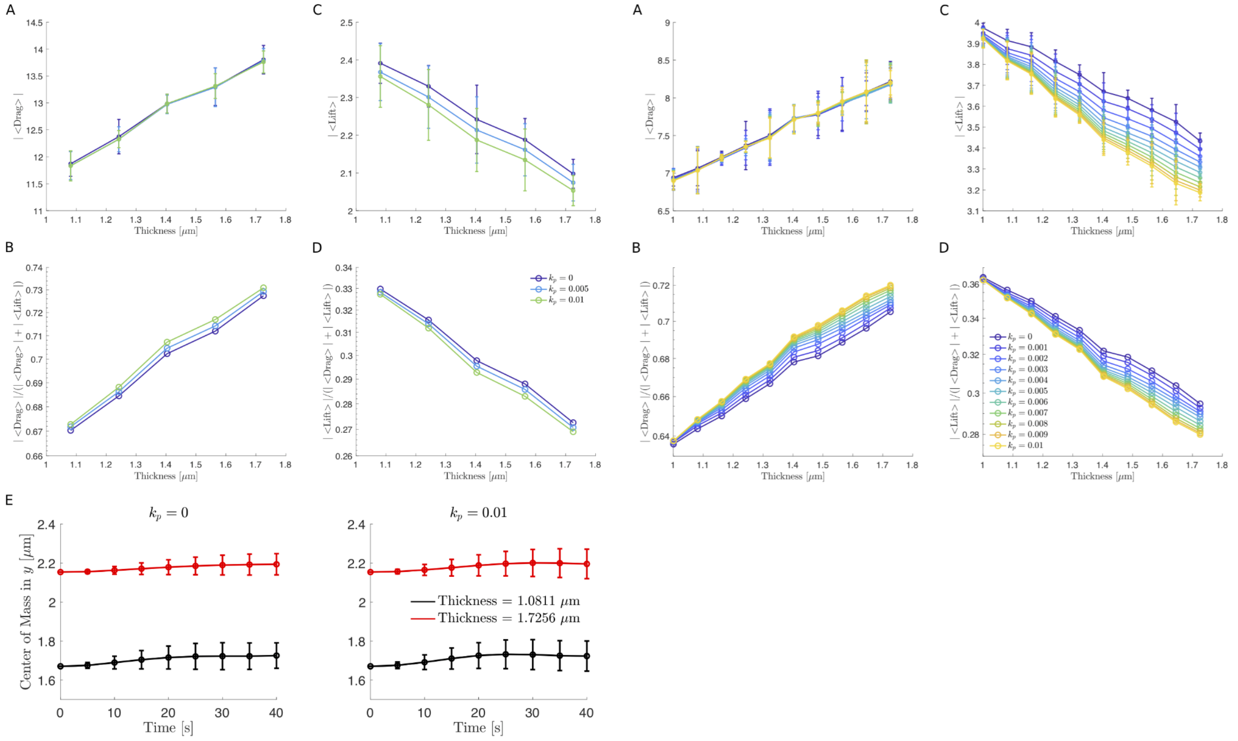

To address the reviewer's comment regarding the experimental study, we agree with the reviewer that the change seen in sepsis might be largely due to vessel narrowing. Yet here changes in vessel wall geometry are caused by disturbed ESL, which leads to leukocyte adhesion and thus narrowing the vessel. In Fig 14 (also attached on the top row below) we have repeated the RBC only simulations using straight channels with width corresponding to the average width of the healthy vessel and the septic vessel. We find that the width of the CFL increases as the width of the channel is decreased. Although the relation between the channel width and the width of the CFL is similar to that of using the extracted vessel wall geometry, the width of CFL is much less uniform across the whole vessel using experimental data, shown on the bottom row below (see lines 395-399).

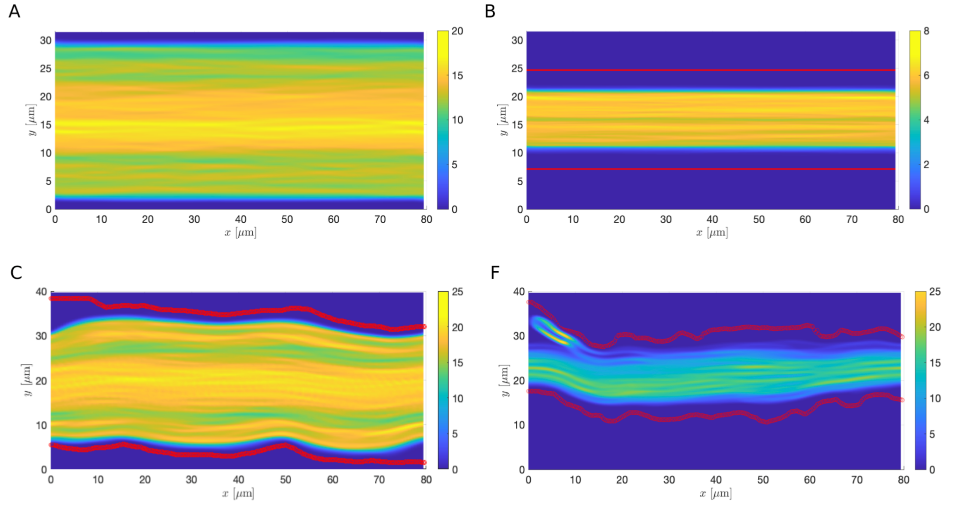

Another confounding factor is the implementation of uniform flow. It is difficult to tell if this is the right choice for effective boundary conditions. Thicker ESL will increase flow resistance. If we assume constant pressure drop, flow will decrease, and cell migration will be altered in one way. If we instead assume constant flow and cell migration will not change due to decreased flow. I am not sure which of these two is effectively implemented (or what combination) and how that may affect cell migration. It seems that if we increase ESL thickness, the constant body force plus increased vessel resistance will affect the flow but I'm not sure how. This is an additional factor that could affect migration and clarification or use of a more standard boundary condition may be helpful. The red blood cell selection process for the septic case may also be playing a role in outcomes. For the septic case, the selection procedure seems to be biased towards lower percentages of red blood cells which produce wider cell free layers. Either more justification or an alternate procedure that can be justified (based on upstream and downstream expected behaviors) would probably improve the study.

Response:
In this work, we applied a sinusoidal body force to initiate blood flow, which has the same effect specifying the pressure drop. Given the specified pressure drop, increasing the ESL thickness (i.e. decreasing the vessel diameter) decreases the blood flow. As a validation, we have simulated the flow (without RBCs and ESL) using varying channel diameter and find that indeed the flow velocity decreases with the channel width.

We agree with the reviewer that the RBC initialization procedure implemented in this work gives a lower percentage of RBCs. However, the concentration of RBCs is expected to be lower in narrowed blood vessels as the concentration of red cells decreases with decreasing vessel diameter [7,8]. We have added two additional references [7,8] along with a brief explanation to further justify our initialization of the RBC's for the septic case (see lines 336-338).

For counterintuitive results, decreasing the vessel width typically enhances lateral migration of red blood cells. Increasing the ESL thickness would, most would expect, effectively decrease the vessel width. Yet the results reported suggest less lateral migration. This result needs further explanation/discussion in terms of why this might happen. Additionally, the fact that this is counterintuitive and why it is should included in such a discussion.

Response:
We agree with the reviewer that lateral migration is enhanced in narrower channels but in a relative sense. That is, the ratio of CFL thickness to vessel ratio increases with decreasing vessel diameter, as shown in Fig 15 of the revision, included here for convenience. Our results are consistent with this finding.

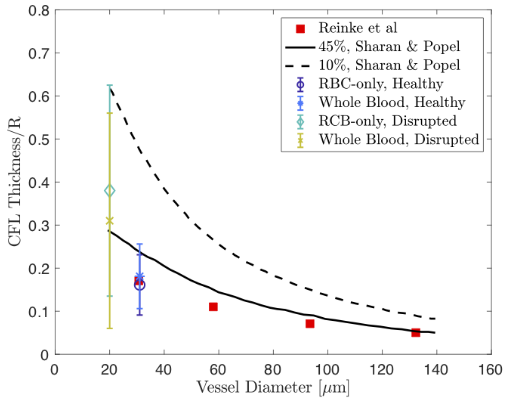

We note that how the lateral migration and lift force are related is subtle and not straightforward. In particular, note that in this work, we are implementing a boundary condition corresponding to a pressure drop. With this set up, as the vessel width decreases the hydraulic resistance increases and thus reduces the blood flow velocity. The slower fluid velocity will lead to a decrease in the lift force for the RBC, which is consistent with the result we obtained from simulations. Therefore, lateral migration is a result of competing effects. Moreover, in the presence of multiple cells, the lateral migration law also depends on factors such as cell-cell interactions. This is beyond the scope of this work but would certainly be an interesting future direction. We have included these points in the Discussion on p. 20.

The non-dimensionalization is confusing. The authors state they are using a 1x2 nondimensional domain but later dimensionalized pictures suggest either the impermeable domain has a different aspect ratio than the permeable domain or there is a different effective domain mesh spacing being used between the two, not just one as listed in the parameters table.

Response:
To avoid further confusion from non-dimensionalization and make the manuscript more consistent throughout, we now report the results in a dimensional manner.

Lines 10-14. Red blood cells migrate even in Stokes flow (see above) where lift force is zero. In addition, their migration is believed to be a cause, not an effect ("Upon formation of the CFL, RBCs …migrate away from the wall"), of the cell free layer. I suggest revising these lines.

Response:
We thank the reviewer for pointing it out. We have revised the sentences to indicate that the formation of the CFL is a result of the wall-induced migration of RBCs. "One important physical phenomena resulting from the wall-induced migration of RBCs is the formation of a cell-depleted layer or cell-free layer (CFL) near the wall [36,37,40]" (see lines 18-20).

Lines 18-19. The comment regarding resistance is a fundamental one deserving citation to an authoritative source. Perhaps one of the Pries+Secomb studies might work?

Response:
We thank the reviewer for pointing it out. In the revised manuscript we have reorganized the paragraph and added a reference [33] as an authoritative source (see lines 25-27).

Figure 1: Having a dotted line in a different color for the ESL “fluid model” boundary would be helpful

Response:
Fixed. We switched to a blue dotted line to show the top and bottom boundaries for the fluid model.

Equation 6: kb, not ks

Response:
Fixed.

Line 95-96: “we … model the ESL as an immobile structure” followed by “we model the layer as an elastic structure”. Immobile sounds rigid, not elastic, is that what is meant by immobile in this context?

Response:
We have changed it to “model the ESL as an elastic structure that is not diffusing with the blood flow” on p. 5.

Equation 14: How thick is the ESL? Probably should be mentioned here. I found ESL = 1 micron in a later section but not in other sections (see above).

Response:
We believe the reviewer is referring to the denominator of Equation 15 and 16. ESL thickness varies in this study – it is fixed to be 1.5 micron for investigating the effect of spatial variation with changing permeability, whereas it is changing from 0.8 micron to 1.8 micron for investigating the effect of thickness with changing permeability. Eq 14 provides the formula for how we computed the ESL velocity in the immersed boundary method setting.

“[0,2]x[0,1] unless otherwise stated”-was this stated differently elsewhere? I feel like this is the only place where dimensionless numbers are used. Maybe just eliminate them (and use

standard Navier-Stokes)?

Response:
We have taken the reviewer's advice to heart and eliminated the dimensionless units.

Line 195-96: In general, there is difficulty with reproducibility in this paper. This is one example. I encourage the authors to put this expression in the appendix, for better reproducibility.

Response:
We have added a new subsection "Biconcave-shaped RBC" at the end of S1 Appendix that gives the parametric form of the equations for generating the biconcave-shaped RBC.

Line 199: "at least 5 fibers". How many exactly? Again, reproducibility.

Response:
We have fixed the text, it should just be "5 fibers" (see line 212).

Lines 208-209: "model stiff fibers". It sounds like "stiff fibers" correspond to the structural components of the ESL and not the "fibers" that make up the membrane of the RBC. I think it should be stated. It says here that the ESL fibers are tethered but in other places both before (line 98) and after (line 458) that they are free.

Response:
For Lines 208-209 (now lines 222-223), we have changed to "model the fiber components of the ESL". The ESL fibers are not free – the roots of fibers are tethered to the fluid domain boundaries and the rest of the IB points discretizing the fibers are connected to one another.

Lines 234-237. It appears that increased density increases lift and expected lateral migration/CFL size and that increasing permeability decreases lift and expected lateral migration/CFL size. The way it is stated, it is difficult to tell which trend you are talking about. It feels like you should say each in a separate sentence rather than trying to combine the trends in one sentence?

Response:
We have rephrased the sentence by breaking it down into two separate sentences to explain the scenarios for an impermeable ESL and a highly permeable ESL individually (see lines 254-263).

Figure 5: 95% confidence intervals. It seems like the "four initial conditions" for the cells were used to create these intervals? Probably should mention sooner/in methods section.

Response:
Yes, the reviewer is correct that the confidence intervals are generated using the sampled initial conditions, as we now mention in the first result section when we introduced the drag and lift force (see lines 244-245).

Line 287: Description of the experiments and, for instance, how exactly the ESL is estimated is missing from the methods section. True, this has been covered in a different paper, but I think readers would appreciate if this info was in this manuscript (though significantly abbreviated) as well.

Response:
We have included a brief explanation based on the information provided in [53] about how the technique used to take the images and how the ESL is measured using the images. "The ESL is measured by calculating the distance between the erythrocytes and the endothelium using the intravital microscopic images [53]" (see lines 325-327).

Lines 299-300: Not sure what's trying to be said here. "While we try to extract the ESL boundary for the septic case"?

Response:
We have rephrased the sentence as "Therefore, we manually trace the boundary of the blood vessel, including the adhered leukocytes, to extract the vessel wall geometry" to clarify our protocol of extracting the ESL boundary from the image corresponding to the septic case (see lines 329-331).

Line 326: Probably should cite the choice of 6 microns for the radius.

Response:
We have added two references [2,3] for supporting this choice (see line 362).

White blood cell study: I believe white blood cell distributions tend to be higher in larger vessels and lower in smaller vessels. The authors should check into this before assuming white blood cells are distributed uniformly throughout the microvasculature.

Response:
For the simulations including leukocytes, for the purpose of comparison, we use the same leukocyte density in both cases. However, these are isolated blood vessels subject to a specified pressure drop; we do not assume that the white blood cells are distributed uniformly throughout the microvasculature. In the revised manuscript, we have added a note with

supporting citation “Although here we assume the same leukocyte density in the healthy and septic scenarios for purposes of comparison, the leukocyte density is known to vary through the microvasculature [27]” (see lines 365-367).

Figure 10: Doesn’t seem to add much to the discussion. Is there something I’m missing about it?

Response:
Figure 10 (now Fig 15) provides a comparison of the CFL thickness to those reported in literature, serving as a validation for showing that our simulation results align with some of the previously reported results. In particular, it compares the CFL thickness estimated from our simulations to simulations of blood flow in narrow tubes from Sharan and Popel [Sharan, M. and, Popel, A.S. (2001). A two-phase model for flow of blood in narrow tubes with increased effective viscosity near the wall. *Biorheology*, *38*, 415-428] and experiments provided by Reinke et al [Reinke, W., Gaehtgens, P., and Johnson, P.C. (1987). Blood viscosity in small tubes: effect of shear rate, aggregation, and sedimentation. *Am. J. Physiol.*, *253*, H540-H547].

As opposed to the lift, which is a more local measure of the wall-induced migration, the CFL thickness quantifies the wall-induced migration through the separation of blood constituents, which is believed to have important physiological roles e.g. on platelet and leukocyte margination.

2D vs 3D model. 3D simulations are feasible nowadays with Karniadakis group making algorithms that can run hundreds of millions of cells and Adrianna Gillman running hundreds of cells in hours (I believe) on her laptop. Developing the algorithms may take some time and 2D simulations may be easier in that respect. 2D simulations are also easier for other reasons as well, but I feel the authors claims regarding computational feasibility for 2D vs 3D are overstated.

Response:
We agree with the reviewer that the two-dimensional (2D) simulations are an important limitation of the model. We justify the use of a 2D model in the present work by the relative computational tractability of 2D simulations. Obtaining the steady-state distributions of red cell and leukocytes through the capillary requires sufficient statistics and relatively long simulation times, and given the significant computational challenges of simulating fluid-structure interaction in three dimensions, performing the analogous simulations in 3D requires faster methods and further computational resources. We believe that the present study provides good evidence that the qualitative behavior found is robust and likely to persist in 3D. However, future studies in 3D are needed to conclusively demonstrate these trends and to obtain more quantitative results. We have extended the Discussion to discuss these reasons for using the 2D model as well as its limitations (see lines 447-454).

Lubrication effect: For tube flows it doesn't seem like the cells should ever get within 4 grid points of the walls. It also seems like your experimental simulation also didn't require lubrication elements. Was there another application in mind?

Response:
As the reviewer suggests, in our case the standard IB method is sufficient for the study as the cell is initialized *not* too close to the boundary – there are at least 4 grid points between the cell and the boundary. Therefore, to avoid any potential for confusion we have removed the remark on incorporating lubrication effects from the revision.

Why unsteady vs steady Stokes flow?

Response:
The unsteady Stokes is commonly used in studying flow with low Reynolds number [9,10,54]. The main reason for this is that it is convenient numerically since the matrix to be inverted involves a perturbation of the identity matrix. That is, with the inertia term that appears in the unsteady Stokes equations, we obtain a well-conditioned linear system. In the main text (lines 124-126) we have added the indicated references above to further clarify our choice.

Line 450: What is phi? Again, I would put it here for reproducibility (there are shorter ways to write it than the way Peskin writes it).

Response:
In S1 Appendix, we have explicitly written out the standard 4-point delta function used in this study, see Eq. 21.

Eq. 26: Should say more. Seems to be for small deformations and no reference curvature.

Response:
We are following the approach in [52] where a bead-and-spring model is used with nodes connected by Hookean springs and the spontaneous curvature is set to be zero and not for small deformations. Together with the area conservation energy with large area preserving constant, this ensures that the total area and the perimeter of the RBC is a constant, which aligns with the scenario in reality. We have included the reference and a brief explanation in the subsection "Discrete energy functions" in S1 Appendix to clarify our choice and the model set up.

Eq. 27: This doesn't seem correct. If the cell is far from the origin, the magnitude of the cross-product will always be positive and large giving a very large sum for the "current area"

compared to the reference area A0.

Response:

We thank the reviewer for catching this typo. The area is calculated by summing up triangles and so the cross-product should be computed using edge vectors. The origin moves as the cell moves and therefore, the magnitude should not be changed. We have fixed the equation, now Eq. 28.

Reviewer #2:

The paper is interesting and well-written. I would like to see some explanation in the variation of lift and drag forces from a fluid-mechanics point of view when the permeability is varied. I found some aspects of the paper that need to be clarified before publication (explained in the comments below).

Response:
We thank the reviewer for their positive comments on our work. We clarify the points detailed by the reviewer in the comments below.

Major Comments:

1. Please provide more biological background and motivation in the introduction. Why is wall-induced migration and maintaining the cell free layer biologically important? Please comment on changes in the ESL during sepsis in the introduction and what causes these changes.

Response:
We have taken the reviewer's feedback to heart and improved the manuscript by including a more thorough discussion of the biological motivation. We significantly expanded the introduction and included more detailed explanations for the biological motivation, studies done previously and our specific contributions to the problem.

How the ESL changes during sepsis and causes is still an open research question. In the introduction we have briefly discussed the relevant studies, "Previous studies on mice reveal that mice lacking the TNF receptor 1 were resistant to heparanase and an enzyme that degrades heparan sulfate, which activates ESL degradation and thus decrease the permeability of the layer [23,24]. Shedding of the ESL occurs in the presence of oxidants, hyperglycemia, cytokines, and bacterial endotoxins, which is closely associated with sepsis. Shedding of the ESL leads to a sparser distribution and structural changes of the vessel geometry mainly in an increase of thickness resulting from leukocytes sticking to the layer [25,26]" (see lines 105-111).

2. What is causes lift forces and wall-induced migration? Is this just a consequence of channel flow (fastest in the middle) that an object moves away from the walls? Please explain.

Response:
In the absence of inertia, the causes of the lift and the wall-induced migration are the interactions of the deformable object with the non-uniform shear rate in the channel flow. The presence of the solid wall near deformable cells introduces an asymmetry in the forces on the cells, which lead to this wall-induced migration. Previous studies have shown that the

generation of lift forces is due to asymmetry of the cell shape or orientation. We also expanded the introduction to explain the cause and consequence of the generation of the lift force and the wall-induced migration in greater detail (see lines 10-18).

3. When the layer is more permeable, does the flow become more or less laminar? I wonder if there is a loss in vorticity that is leading to the changes in lift forces. Perhaps the authors could compare the vorticity at different values of permeability, even in the simplified version of the model. It seems like the velocity boundary conditions are no penetration in the normal direction and nonzero in the tangential.

Response:
In the first part of the manuscript in which we use a single RBC to study the influence of the ESL, we find that permeability plays a minimal role (see Fig 6 and Fig 11, also attached below) and does not affect the flow significantly.

Fig 6 (The difference between the maximum and minimum values of the drag and lift ratio versus permeability using the microscopic ESL model):

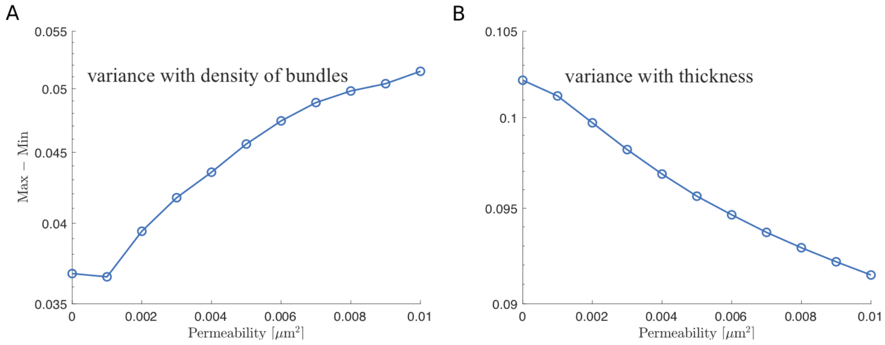

Fig 11(The difference between the maximum and minimum values of the drag and lift ratio versus permeability using the macroscopic ESL model):

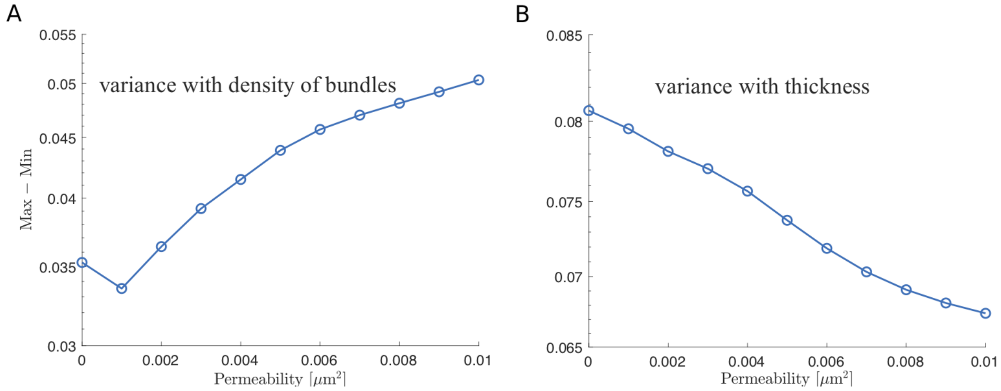

To further illustrate this, we took the detailed model and examined the vorticity of the flow for two extreme cases when the ESL is impermeable (k_p = 0) and highly permeable (k_p = 0.01) at steady state. The following vorticity plots do not demonstrate a notable difference in the two

cases.

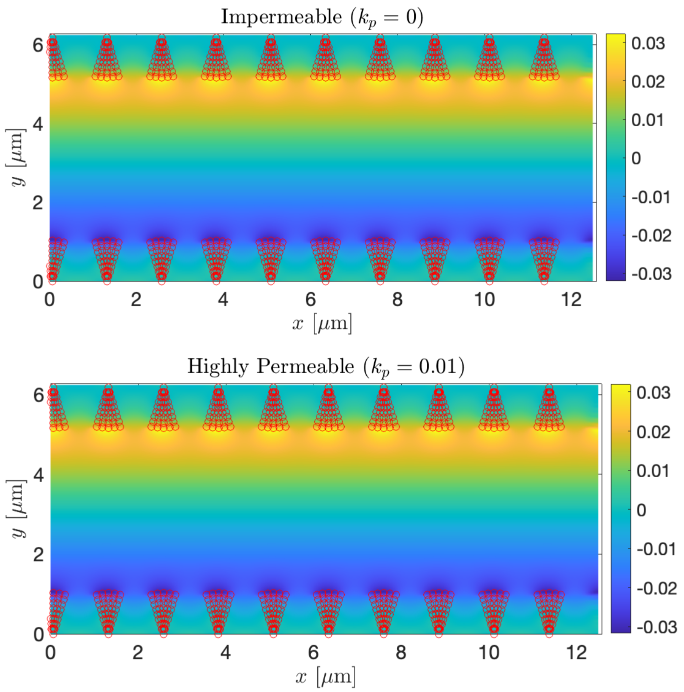

4. In the discussion of Fig. 4 (lines 220 - 240), I found it a bit difficult to see the variation in life and drag as a function of bundle density for each value of permeability. Perhaps the authors could add a figure showing the maximum minus minimum values for the data in Fig. 4 C and E as a function of permeability to highlight the variation in lift and drag (similarly for Figs. 5 - 7).

Response:
To further support the discussion of the change in the lift and drag ratio as the spatial variation and the thickness of the ESL is varied for both models, we have generated two additional figures showing the (max-min) versus permeability (see Fig 6 and 11, also attached below). Note that since the relative drag (Drag/(Drag+Lift)) and relative lift (Lift/(Drag+Lift)) sum up to 1, the (max-min) remains the same for both the relative drag and relative lift. Therefore, we only plotted the relative drag to demonstrate the variance.

Fig 6 (The difference between the maximum and minimum values of the relative drag versus permeability using the microscopic ESL model):

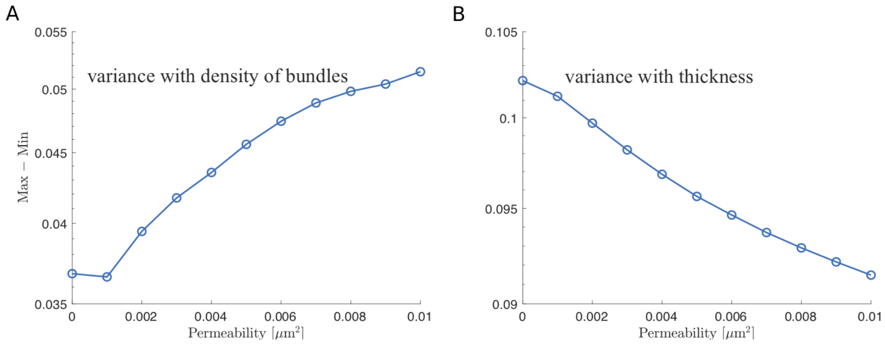

Fig 11(The difference between the maximum and minimum values of the relative drag versus permeability using the macroscopic ESL model):

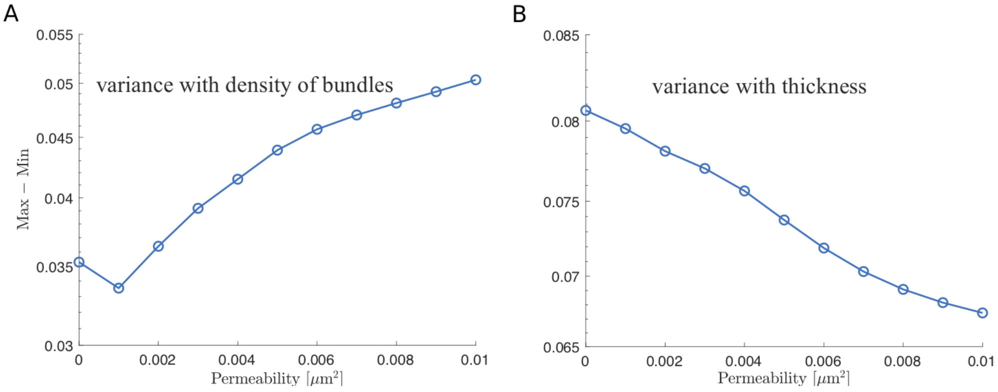

Also, in this section, if the lift forces are increased, is the cell more likely to remain in the middle of the channel? It would be helpful to see a representative graph of the position of the cell in the channel for a case when the lift forces are low versus high.

Response:

In response to this comment and similar ones from Reviewer 1, we have included 4 figures showing the positions of the RBC for selected cases (see Fig 2 B and C, Fig 4, Fig 7 B and C, and Fig 9, also attached below) to motivate and support the analysis of the drag and lift force. In the case when the lift force is low (i.e. black curves in Fig 2 and Fig 7), it takes the RBC a longer time to move to the centerline compared to the scenario when the lift force is high (i.e. red curves in Fig 2 and Fig 7).

Fig 2 B and C (The distance of the RCB's center of mass to the wall for different spatial bundle density):

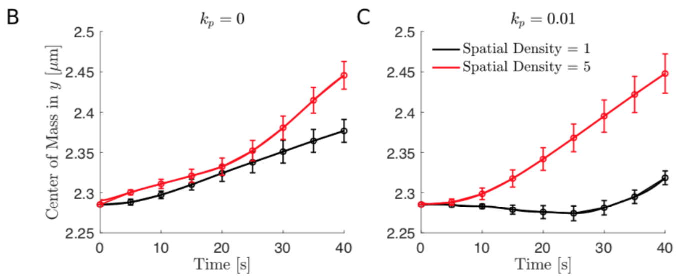

Fig 7 B and C (The distance of the RCB's center of mass to the wall for different spatial frequency using the macroscopic ESL model):

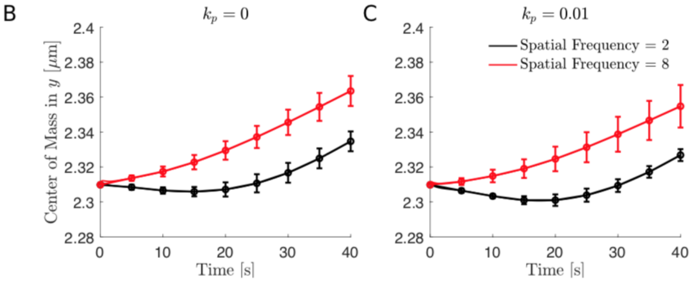

Minor Comments

1. Line 30, In the introduction, the authors state that osmotic pressure variation was used to determine the endothelial surface in elastic. How can osmotic pressure be used to determine this? How much deformation occurs in the surface layer?

Response:

We have simplified the sentence and removed "through analyzing the variation of the osmotic pressure" to avoid potential confusion as it is not the focus of the present work.

2. On Line 59, the authors refer to a disrupted scenario, but they haven't defined it at this point in time. Is the disrupted scenario the narrowed channel geometry in Fig. 8E?

Response:

Yes, the reviewer is correct that here the disrupted scenario refers to the septic case as shown in Fig. 8E (now in Fig. 12E). We have replaced the word "disrupted" now in lines 103-117 by "septic" to clarify that we are comparing the healthy case to the septic case using the images provided in [53] (previously [31]).

3. In the results section, the authors compare their work to [22]. A brief comparison of the present work to previous the modeling approaches such as [22] would be helpful.

Response:
We have extended the introduction section to briefly discuss other existing modeling approaches. See lines 36-63.

4. Line 89 Eq. (7), why is an area conservation term necessary?

Response:
The area conservation term is necessary as it is known from the newly added reference [1] that immersed boundary methods can leak in the sense that numerical errors may lead to loss of fluid from impermeable membranes, leading to a loss of area. As we are running long-time simulations in which these numerical errors can accumulate to yield significant loss of enclosed area, it is necessary to implement such an area conservation energy to avoid loss of area. We have added a sentence clarifying the reason for including the area conservation energy (see lines 147-150).

5. On p. 2, line 41, the first model refers to the course-grained model, but on p. 4, line 96, the first model refers to the detailed representation of the ESL. Please choose one model as the "first".

Response:
We have eliminated “first” and “second” in the text and now use “microscopic ESL model” and “macroscopic ESL model” throughout the manuscript.

6. In both models, does the ESL have internal elasticity or just tether forces? The discussion on pp. 8-10 seems to contradict the description on p. 5 (line 98).

Response:
In the macroscopic ESL model, only tether forces are used. In the microscopic ESL model, both elastic and tether forces are implemented. The description of the ESL elasticity has been clarified in the revision on the bottom of p. 5 and the top of p. 6.

7. On p. 6, line 138, the authors state that they "calculated both forces by averaging over four initial conditions that either uniformly sample the distance between two neighboring bundles for the detailed model or one wavelength of the sine wave for the coarse-grained model." How are these 4 initial conditions different? I had a difficult time determining this from the text. Was the orientation of the cell fixed at 30 degrees in each case? Please clarify.

Response:
The reviewer is correct that the orientation of the cell is fixed at 30 degrees for all 4 initial conditions. It is the position in the x direction that is changed. To illustrate we have added a

reference to the appendix figure (S5 Fig) showing an example of the 4 initial conditions.

8. On p. 8, line 186, is the biconcave shape just an initial condition? Did the authors add a reference curvature or use a different energy functional maintain this shape? Please explain.

Response:
The biconcave shape is used as the initial condition/configuration and it is not forced to be maintained through the simulation. In the absence of flow, the biconcave disk shape is maintained because it minimizes the bending energy subject to the perimeter and enclosed area constraints. We have added a new appendix section providing the parametric equations used in this work to generate the biconcave initial shape (see lines 532-536).

9. On p. 10, line 248, was the height of the bundles increased to model a thicker ESL? Please clarify.

Response:
Yes, the reviewer is correct that in the microscopic ESL model, the height of individual ESL fiber bundles is also changed. We have added a sentence "To determine the importance of the ESL thickness, we assume the density of bundles is fixed to 5, and increase the height of individual ESL fiber bundles, h, with values given in Table 1" on p. 10, line 270 to clarify.

10. On p. 15, Fig. 8, which value of permeability was used for these simulations? Table 3 shows a range of values.

Response:
For simulations shown in Fig 8 (now Fig 12 in the revision manuscript) we assumed that layers are impermeable, $k_p = 0$. This originally appeared in the caption of Fig. 8 – the sentence "Each ESL is assumed impermeable ($k_p = 0$)".

11. I am confused about the different between healthy and septic with the extracted geometry. Is the main difference the narrowing of the channel?
It seems that the ESL is not modeled as a permeable structure for the whole blood simulations. Does the ESL act as an elastic no-slip boundary?

Response:
The extracted ESL geometry in the healthy and septic differ mainly in two places:
1). The thickness of the layer is significantly larger in the septic case and resulted in the narrowing of the vessel as the reviewer pointed out. The change in the ESL thickness is one important factor that contributes to the change in the CFL width as illustrated in Fig 15.
2). The layer is significantly smoother in the healthy case, corresponding to a smaller spatial

variation.

The reviewer is correct that in both the whole blood and RBC only simulations, the ESL is not modeled as a permeable structure. According to the first part of the manuscript in which we use a single RBC to study the influence of the ESL, we found that permeability plays a minimal role in affecting the drag and lift (see Fig 6 and Fig 11, attached on previous pages). For both models used in this work, we found that on average, changes in the permeability leads to changes in the drag and lift that are less than 9%. As such we model the ESL as an elastic no-slip boundary in the whole blood simulations.

12. On p. 14, line 317, how exactly is the cell free layer measured? Is it an averaged distance from the red curve to where the steady state distribution is nonzero in Fig. 8 and 9? Please explain.

Response:
The procedure of estimating the width of the cell free layer was provided in the original submission on p. 14, lines 314-317 (now lines 347-353). In particular, the width of the cell free layer is measured by averaging 800 vertical slices of the steady-state plots of cell positions, evenly spaced from x = 10 to x = 70. For each ESL (top and bottom) in each slice, we calculated the distance between the ESL and edge of the RBC that is the closest to the ESL. The width of the CFL is then obtained by summing up the estimated distance in all 800 vertical slices and averaging over the total number of slices.

13. On p. 16, line 333, can the authors comment on why the RBCs move slower in the disrupted case? Is the background channel flow the same in healthy versus disrupted?

Response:
The pressure drop is the same in both the healthy and disrupted case. This leads to a slower blood flow in the disrupted case as the width of the channel is decreased due to a thicker ESL, causing the RBCs to move more slowly. We have included extra sets of simulation results estimating the maximum and mean flow velocity using a straight channel with varying channel width. We see that as the channel width decreases, the flow velocity decreases and thus leads to a slower RBC movement.

14. For the study described on p. 16, last paragraph, do the authors use the extracted geometry when varying the vessel diameter? Please explain.

Response:
Yes, the reviewer is correct. Here we make a direct comparison of our RBC-only and whole blood simulation results using the extracted geometry to those reported in literature to validate our simulation results.
