# Supplementary material for: Influence of the vessel wall geometry on the wall-induced migration of red blood cells
Source: PLoS Comput Biol. 2023 Jul 17;19(7):e1011241. doi: 10.1371/journal.pcbi.1011241 (PMC10374106; doi:10.1371/journal.pcbi.1011241)

Dear PLOS Comp. Biol. Editor,
Thank you very much for your consideration of our manuscript. We thank the reviewers for their helpful comments and suggestions. Major changes to address the reviewers comments include:

1. Changing the title of the manuscript to “Influence of the vessel wall geometry on the wall-induced migration of red blood cells”.
2. Significantly re-editing the Abstract, Introduction, Result, and Discussion sections to clarify that we are investigating a special case where the vessel wall geometry is changed by the ESL under different pathological conditions.
3. Adding new simulation results (figures and movies) for the septic case using the same hematocrit as in the healthy case.
4. Redesigning the CFL study more systematically and comparing our results to the literature.
5. Adding a new Appendix section to discuss the mobility tensor in 2D.

Please find the attached updated manuscript with changes in blue. We have provided detailed responses to the reviewers’ comments below and in the updated text.

Sincerely,

Ying Zhang and Thomas Fai

Reviewer #1:
The reviewer thanks the authors for their work on the most recent manuscript. The reviewer appreciates the several clarifications that have been made and still appreciates the availability of their work, its scope, and the time and effort that it took. Still, the reviewer is concerned that many suggested changes were only minimally addressed and/or made in the manuscript and encourages the authors to look over the reviewer's previous suggestions more carefully/again.

Response:
We appreciate the reviewers' positive feedback as well as the reviewer's help to make the manuscript better. We have taken the comments and suggestions to heart and done our best to address them, as we discuss below.

Major comments:
One major concern is that there are multiple implied statements that the authors seem to make that are not exactly canonical. One example, which I already commented on previously, is that the in vivo study implies to the naive reader that the ESL thickness actually increases to 6-10 microns because during sepsis because the ESL entrains/entraps multiple immune cells. The studies I have read state that the ESL is considered to be a layer of various molecules and they never include cells in the layer. One sentence is included on how disrupting the ESL is believed to bring in immune cells and cause narrowed vessels. The rest of the manuscript, however, reads as if disruption increases thickness and narrows the vessel without any reference to the immune cell role.
Response:
We appreciate the reviewer's feedback and have clarified our terminology to delineate between changes in the ESL and other influences on the vessel geometry. In particular, we have clarified that while the ESL damage that occurs in pathologies such as sepsis may lead to narrowed blood vessels via adhesion of blood constituents such as leukocytes, it is not actually a change in ESL thickness but rather the adhesion of leukocytes that causes the narrowing of the vessel (see lines 127-130). We have accordingly changed the title to "Influence of the vessel wall geometry on the wall-induced migration of red blood cells" and highlighted in the manuscript that we are focusing on a special case in which changes of the vessel wall geometry are induced by ESL.

Another similar worrying statement is that decreased CFL leads to increased blood velocity. This is entirely against canon as the CFL (I'm fairly certain) was originally

hypothesized to explain why blood was going faster than expected, a wide CFL was needed to explain faster, not slower, velocities. Related, we have an extreme change in hematocrit from 45% to 10%. Based on this alone, it is no surprise to me that the CFL is much wider in the low hematocrit scenario than in the other.

Response:

We regret any confusion, and have clarified our claims regarding the influence of CFL on blood velocity, which we show to be consistent with the literature. According to the results in Refs [41, 42, 43] where the blood vessel is represented by a *tube*, we can draw the following conclusions:

(a). For a fixed hematocrit, the CFL increases as the vessel diameter is increased and the blood velocity is faster.

(b). For a fixed vessel diameter, the CFL increases as the hematocrit is decreased and the blood velocity is faster.

How the CFL and blood velocity correlate with each other depends on many factors such as vessel diameter and hematocrit. To make the study more systematic and avoid potential confusion, we have made the following changes:

1. We first re-simulated the septic case using the same hematocrit as in the healthy case for both the RBC-only and whole blood cases (see Fig.12 and 13, also attached below). In both cases, we found that the width of the CFL is decreased and RBC velocity is slower in the septic scenario as shown in the table below. Our results completely align with bullet point (a) summarized above.

		Mean RBC Velocity [mm/s]	Top CFL [μm]	Bottom CFL [μm]
RBC-only	Healthy	0.528	3.316	1.101
	Disrupted	0.223	2.29	1.013
Full Blood	Healthy	0.444	3.705	1.1
	Disrupted	0.221	3.63	1.084

Fig.12 (RBC-only simulations with extracted vessel wall geometry using the same cell density in both cases)

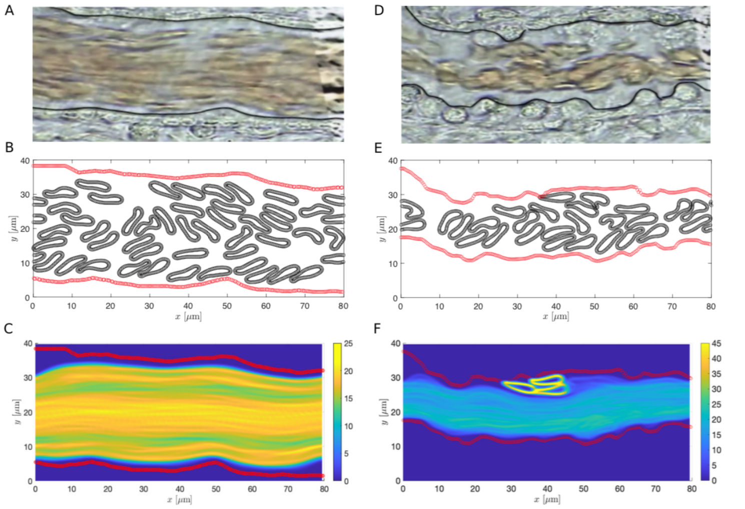

Fig.13 (Whole blood simulations with extracted vessel wall geometry using the same cell density in both cases)

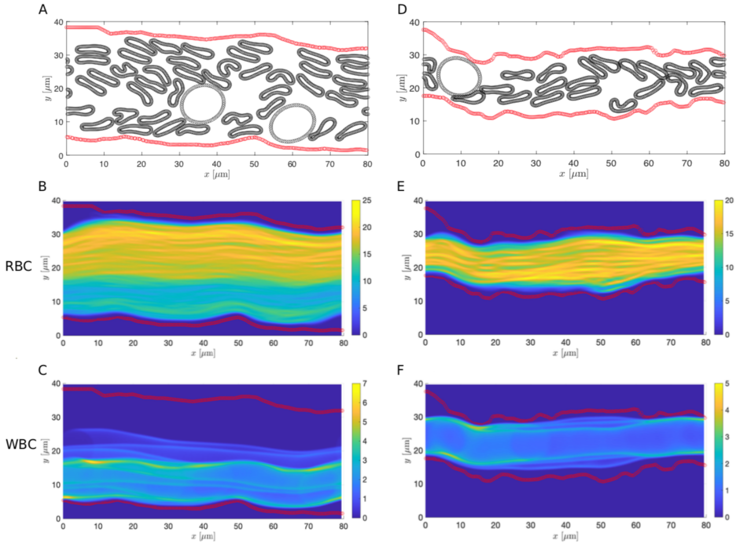

2. Next, we compared the newly simulated results to the ones that use a lower hematocrit (see new figures Fig.14 and 15, also attached below). We found that the width of the CFL is increased as the hematocrit is decreased and the mean RBC velocity is faster as shown in the table below. Again our results are consistent with literature (bullet point (b) above).

		Mean RBC Velocity [mm/s]	Top CFL [μm]	Bottom CFL [μm]
RBC-only	Disrupted (High Density)	0.223	2.29	1.013
	Disrupted (Low Density)	0.324	3.633	3.99
Whole Blood	Disrupted (High Density)	0.221	3.63	1.084
	Disrupted (Low Density)	0.258	3.289	1.896

Fig.14 (Comparison of the RBC-only simulations in the septic case using different cell densities)

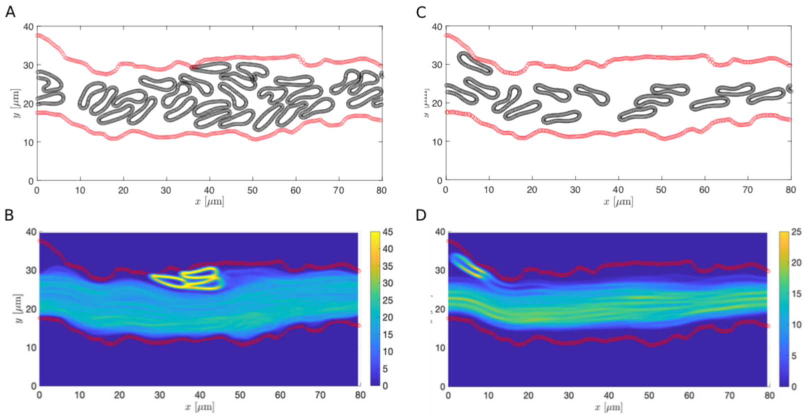

Fig.15 (Comparison of the whole blood simulations in the septic case using different cell densities)

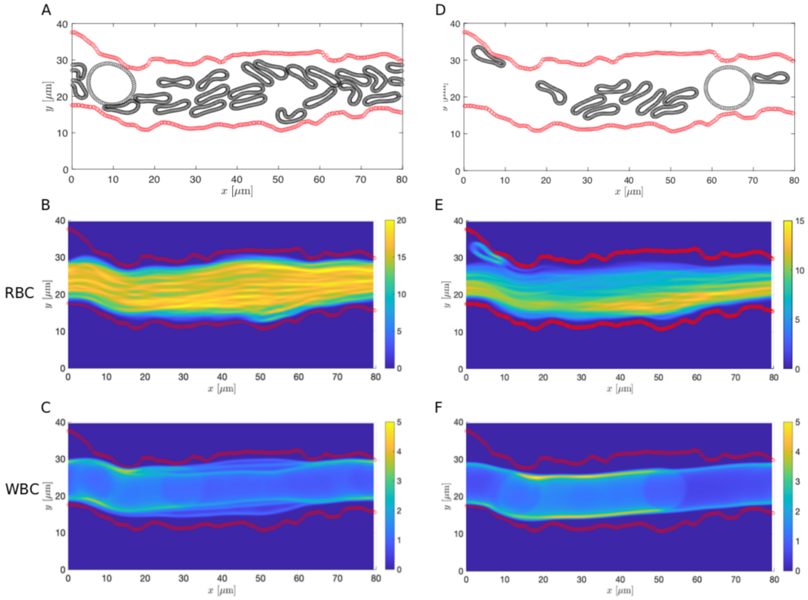

3. We updated Table 4 to incorporate all the information mentioned above.

These issues all stem back to confounding factors. When multiple things change at once, it is difficult to know which is responsible for the effects seen and can lead to false (implied and/or perceived) claims such as how the ESL gets microns thicker during sepsis. I encourage the authors to try to avoid changing everything at once, as they do in their last study, and instead develop a more systematic study that changes things one at a time. E.g. with/without white blood cells, with same vs different hematocrit, with same/different velocities, with same/different widths, etc. Saying that "lateral migration is a result of competing effects", while true, does not help us identify which effects are more or less important.

Response:

We appreciate the reviewer's suggestion and have reorganized the section “Cell-free layer estimation for healthy and disrupted ESL” with changes mentioned in the response to previous comments that involve systematically changing system variables one at a time (see above).

Minor comments:

Please define "mobility". What is the actual tensor? (Perhaps in supplementary info.)

Response:
We have added a new section “S6 Appendix. The mobility tensor” providing the definition of the actual tensor (see pages 27-28).

To make the macro model more obvious, one should probably name and label in Figure S5 the fibers for both micro and macro models (e.g. 9-10 does a little bit of that).
Response:
We thank the reviewer for the suggestion. As explained in the figure caption, the red dotted curves correspond to the ESL layout in the two models. To clarify further, we have added the labels “microscopic model” and “macroscopic model” next to the red curves in both panels (the updated figure is also attached below).
S5 Fig (Schematic of the ESL layout and the initial positions of the RBC)

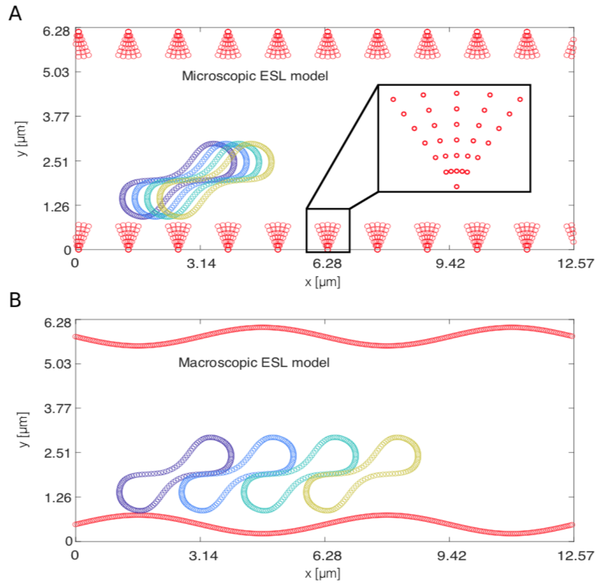

For equation 6, I'm guessing the energy is meant to be curvature squared as it is in the Helfrich bending energy with zero reference curvature. If that's the case, I'm pretty sure the curvature expression used in equation 6 is for small deformations (see Stewart Calculus, for instance). When q corresponds to the current arc length, the expression yields the curvature but if q corresponds to Lagrangian coordinates that can be significantly stretched and differ from the geometric arc length, the expression does not yield the exact curvature.
Response:
The reviewer is correct that this represents a Helfrich-type bending energy with zero spontaneous curvature, following the approach in [52] where a bead-and-spring model is used to describe RBC membrane in 2D Poiseuille flows. Regarding whether the curvature expression is exact or approximate, we note that our formulation also includes

a stretching energy, so that the deviation between the Lagrangian coordinate q and arclength should be small. As a validation, we measured the maximum difference between the total arclength in the deformed and rest configurations of the RBC using both the microscopic and the macroscopic ESL model over time. In both cases, the maximum difference calculated from simulations is less than 3%. We have included a short sentence to address this in the manuscript (see lines 172-176).

To further justify our choice of the RBC membrane energy, we have included three new references [88,89,92] (see line 159) where the same stretching and Helfrich-type bending energies are used.

There are several typos/grammar issues throughout. Unfortunately, there seem to be more in this version of the manuscript than there were previously. Fixing this, I expect, will make the manuscript read more smoothly.
Response:
We thank the reviewer for pointing this out. We have done a careful read-through and fixed the typos/grammatical errors.

There seem to be introductory paragraphs in some of the Results subsections with material that probably belongs in the introduction.
Response:
We thank the reviewer for the suggestion. We have done a careful read-through and edited the text in the Introduction and Results sections accordingly. For instance, we have moved the majority of the introductory paragraph in the CFL section to the Introduction.

The hematocrits chosen. If one has information about the expected hematocrit as a function of vessel size, one should use that to help design one's study. If your healthy case should expect 35% hematocrit while the unhealthy case should expect 20%, then why not use those percentages (while also perhaps taking steps on the way, e.g. comparing 35% in both and comparing 20% in both before comparing 35 vs 20).
Response:
We thank the reviewer for the suggestion and agree that the comparison can be done more systematically without changing multiple things at the same time. We have added new simulation results for the septic case using the same hematocrit. We found that the results are consistent with literature (see response to previous comments above).

The authors have not yet made it clear why drag that goes upstream/downstream only might affect lateral migration. The efforts so far have not been sufficient.

Response:

Lateral migration is an emergent behavior that involves the interactions between cell deformations and the surrounding flow. The shape asymmetry induced by local flow gradients are a direct consequence of the increasing upstream/downstream drag toward the no-slip wall, and the lift on the cell depends on these changes in shape. See lines 105-109 and Refs [5, 71] cited in the manuscript.

"due to the stiffness of ESLs" Is permeability or density meant in the place of "stiffness". I don't recall reading about how ESL stiffness affects flow.

Response:

We have removed this phrase and paraphrased the sentence to avoid further confusion.

Equation 28 is still incorrect as X_i^0 is not explained sufficiently. Why is the 0 there? Perhaps the authors mean "position vector relative to the cell centroid"? By the way, the formula is also problematic/doesn't work for cells that deform too much and lack a star-point. It may or may not matter for forces, but the energy will be off.

Response:

We use X_i^0 to denote the difference between the position vector of the ith Lagrangian point and the position vector of the first Lagrangian point (i = 0). That is, X_i^0 = X_i - X_0. We have included a short explanation for the new notation. For using this energy in modeling RBCs, we have included a reference (see Ref [52]) to support its validity. In addition, we have done benchmark simulations to make sure that the maximum loss of area of each RBC over time is less than 2%, which is stated in the manuscript (see lines 167-168).

Reviewer #2:
The authors made substantial revisions and addressed my comments. I recommend publication after addressing the following minor comments.

We appreciate the reviewer's comments and have addressed the minor revisions below.

On p. 3, line 59, please provide more description of the ESL model from [50]. In particular, the endothelial glycocalyx (EG) appears to be modeled as a chain with elasticity and bending rigidity.
Response:
We have included additional description of the bead-spring model used for the ESL in [50], see lines 71-74.

In Fig. 11 A, the text should be "variance with varying spatial frequency", not bundle density.
Response:
Fixed. The updated figure is attached below.
Fig.11 (The difference between the maximum and minimum values of the relative drag versus permeability using the macroscopic ESL model)

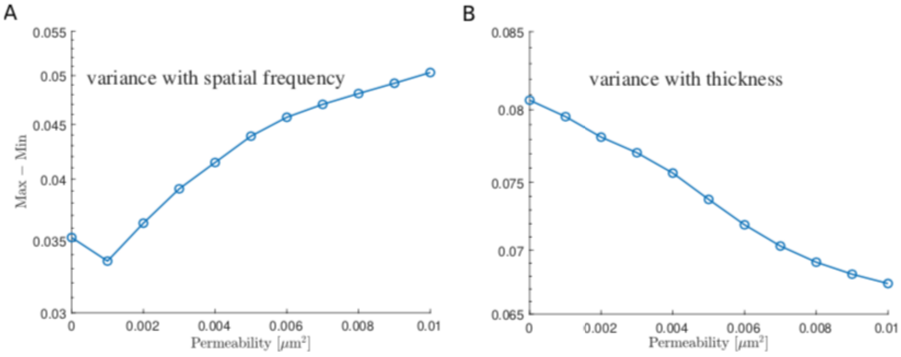

In the new S4 Appendix, the figure references are incorrect. On line 603, I believe S4 Fig should be S6 Fig. On Line 604, S6 Fig should be S8 Fig. Please carefully check the figure references in this section.
Response:
We thank the reviewer for catching the mistake. We have fixed all the incorrect figure references.

On p. 29, the x-axis should be density of bundles to be consistent with Fig. 3.

Response:

Fixed. The updated figure is attached below.

S6 Fig (Effect of spatial variation using a circle in the microscopic ESL model)

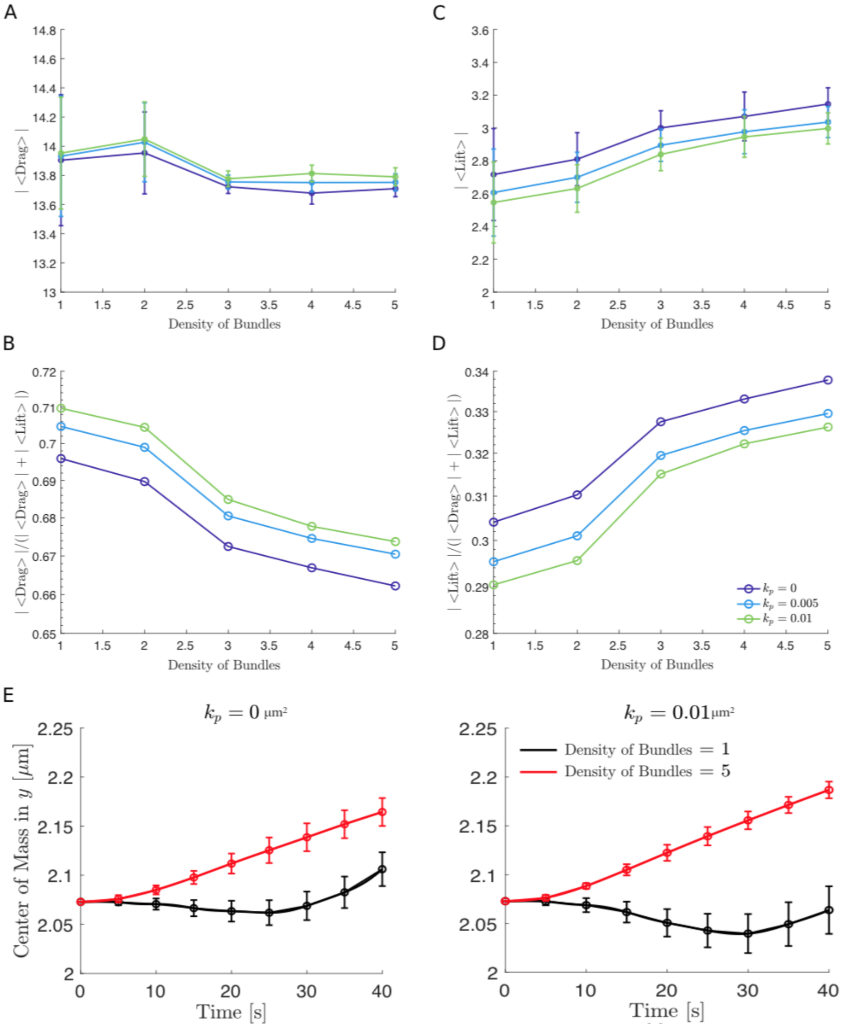

Supplement: Attachment — Submitted filename: Resp_to_rev_PLOS_R2.pdf [file pcbi.1011241.s009.pdf]
