# Supplementary material for: Influence of the vessel wall geometry on the wall-induced migration of red blood cells
Source: PLoS Comput Biol. 2023 Jul 17;19(7):e1011241. doi: 10.1371/journal.pcbi.1011241 (PMC10374106; doi:10.1371/journal.pcbi.1011241)
Supplement: Attachment — Submitted filename: Resp_to_rev_PLOS_R3.pdf [file pcbi.1011241.s010.pdf]

Dear PLOS Comp. Biol. Editor,
Thank you very much for your consideration of our manuscript. We thank the reviewers for their helpful comments and suggestions. Major changes to address the reviewers comments include:

1. Clarify the terminology, in particular the “drag”, used in the manuscript in greater detail.

2. Expanding the Discussion section to discuss the limitation of boundary conditions.

Please find the attached updated manuscript with changes in blue. We have provided detailed responses to the reviewers’ comments below and in the updated text.

Sincerely,

Ying Zhang and Thomas Fai

Reviewer #1:
I appreciate the great deal of work the authors have put into their manuscript including more thorough consideration of many of my comments. I think the manuscript has greatly improved as a result. While I appreciate the more methodical approach that has been adopted by changing factors more strategically, there are a few smaller issues that I am still unclear about.

Response:
We appreciate the reviewers' positive feedback as well as the reviewer's help to make the manuscript better. We have taken the comments and suggestions to heart and done our best to address them, as we discuss below.

Major comments:
The authors comment that "drag toward the no-slip wall" is somehow important for the migration process. This comment and the use of total drag as an indicator for formation of the CFL still confuses me. I feel I need to try my point one more time so that hopefully it's clear to the authors and they are able to address it better. Drag operates up/downstream, not towards the no-slip wall. My understanding is that drag (especially the one plotted in the paper) is the total force operating up/downstream on the cell while lift is the total force operating to/away from the wall. If this is the case (correct me if you are using a different definition of drag), I am still not clear on why total drag continues to be used as some sort of indicator for CFL enhancement and thickness. Is the argument that drag causes asymmetric deformations which causes migration? If so, I'm pretty sure the situation is similar to the story about pressure in incompressible flow, raising or lowering the pressure by a constant does not affect dynamics whatsoever. Only changes in pressure gradient matter. Similarly, changes in the total drag doesn't matter in terms of deformation of cells. Only variations in the upstream/downstream stress distribution across the cell matter for morphological changes. For instance, often there is upstream stress on the portion of the cell near the wall and downstream stress on the portion of the cell away from the wall, causing the cell to deform into slipper-like shapes that move away from the wall. I believe these sort of asymmetric effects is what the cited references discuss. The total drag in such scenarios could be any value, all that matters is the differences in local drag from top to bottom in the cell. If the total drag is, in fact, somehow an indicator of the magnitude of such variation, it should be explained better how that is the case. It doesn't seem to be clear from the references given as they site morphological asymmetries that arise (which I don't think can arise, as argued above, due to total drag on the particle). Both references also seem to consider adhesion which may be giving rise to "insights" not applicable to non-adhering cells. E.g. that adherence mediated "drag" gives rise to more deformation and higher

migration. I encourage the authors to consider this once more, especially since the use of plots of total drag is so prevalent throughout the text, and either make their case stronger than it currently is or to reevaluate.

Response:
We thank the reviewer for pointing this out. In the revision we have clarified how we analyze the lift and drag to study the motion of the RBC. As shown in the newly added reference [93], when the deformable object is moving near a wall, it will experience a larger horizontal resistance as it moves towards the wall. Therefore, when a deformable object is moving near a wall, its motion is tied to its deformation. In particular, we use the lift to assess the ability of the RBC to escape from the vessel wall. We have added a short explanation in the introduction (see lines 101-107) regarding the terminology.

The implementation of permeability still is unclear to me. In particular, is the permeability implemented by applying equations 15 and 16 only at the red locations in Figure 1? Are they also applied at the blue locations in Figure 1? If they are applied at the red fibers, particularly in 1b, it seems like changing bundle density also affects permeability. Is this correct? Clarification on permeability vs. bundle density (vs. thickness) could probably be helpful.

Response:
Yes, the reviewer is correct that the permeability is implemented by applying equations 15 and 16 only at the red locations. Blue locations represent the no-slip boundary for the fluid model as described in figure 1 caption. We agree with the reviewer that changing bundle density and thickness could potentially affect permeability, but how these three factors relate to each other requires further detailed investigation, which would be an interesting future work.

Minor comments/suggestions:
In the abstract, “formation of a cell-free layer” should probably read “enhancement of the cell-free layer” or something similar. There is always a cell-free layer, just its size may be altered.

Response:
We thank the reviewer for pointing it out. We have changed it to “thickening of a cell-free layer”

“which potentially leads to a heterogeneous distribution of RBCs”. Again, RBCs are typically heterogeneously distributed and I suggest “which can increase the heterogeneity of the RBC distribution in capillaries” or something similar.

Response:
Fixed (see lines 26-27).

“although Eq 6” should probably be “although partial^2X/partial^2”. In addition, the rest of the sentence is awkward. Perhaps something like although this does not yield the exact curvature, especially for large deformations, here it differs from the exact curvature by less than 3%.

Response:
Fixed accordingly (see lines 174-176).

181-182. Are tether points at the red locations? The blue locations too? I suppose I was unclear on the blue location in Figure 1. Are those explicitly tracked Lagrangian points or an artist rendering choice?

Response:
Tether points are only applied at the red locations. Blue locations are NOT Lagrangian points and they represent the no-slip boundary for the fluid model as described in Figure 1 caption. We made this artistic choice based on your previous comments regarding separating the fluid boundary from the ESLs. To avoid further confusion, we have added a sentence explaining that the tether points are only implemented to red locations, which correspond to vessel walls (see lines 183-185).

283-4 RBC [migration] is significantly inhibited…caused by a decrease in the drag and an increase in the lift. Increased lift means more migration. I’m not sure why the authors say migration is inhibited in such a case.

Response:
We thank the reviewer for catching the mistake and have fixed the sentence.

RCB vs RBC. Centered of mass vs. center of mass.

Response:
Fixed.

When adding the leukocytes, the authors see the same behavior. Ideally there is at least some difference (e.g., less of a decrease in both quantities) that could be highlighted (or else it would have been sufficient to just show just one of the two cases). Even if there is no {/it significant} difference, that is a result worth stating.

Response:
We thank the reviewer for the suggestion and have added a sentence to state the difference (see lines 389-390).

There are many statements of the sort “they are the same”. This should typically be accompanied by more specifics like, “to within 1%”. This is a good practice in general.

Response:
Fixed.

Discussion of limitations of study should include discussion of boundary conditions. The “correct” boundary conditions for an isolated vessel in a region of sepsis is very difficult to ascertain. It depends on upstream and downstream resistances and comparing healthy and septic scenarios is very difficult because of the uncertainty of those boundary conditions. Depending on the situation, the septic region may receive a significantly decreased or a significantly increased amount of flow. The approach used here is, in addition, somewhat problematic as it is not clear if the approach corresponds to a prescribed velocity or to a prescribed pressure drop, which tend to be the two most well-accepted set of boundary conditions for tube flow. As such, I’m not really sure what this means for where these simulations lie between the expected extremes that might occur in vivo. A discussion of all of this would be appropriate.

Response:
We thank the reviewer for the suggestion. We have added a short paragraph discussing the boundary condition (see lines 513-519).

Reviewer #2:
I recommend acceptance.

Response:
We thank the reviewer for their feedback, which has helped strengthen this work.
